# Conjugation of Polycationic Peptides Extends the Efficacy Spectrum of β‐Lactam Antibiotics

**DOI:** 10.1002/advs.202411406

**Published:** 2024-11-05

**Authors:** Julia Werner, Florian Umstätter, Manuel B. Böhmann, Hannah Müller, Barbro Beijer, Tobias Hertlein, Laura Kaschnitz, Veronika Bram, Christian Kleist, Karel D. Klika, Eric Mühlberg, Gabriel Braune, Sabrina Wohlfart, Martin Gärtner, Silke Peter, Stefan Zimmermann, Uwe Haberkorn, Knut Ohlsen, Heike Brötz‐Oesterhelt, Walter Mier, Philipp Uhl

**Affiliations:** ^1^ Department of Nuclear Medicine Heidelberg University Hospital 69120 Heidelberg Germany; ^2^ Department of Pharmaceutical Technology Institute of Pharmacy and Molecular Biotechnology Heidelberg University 69120 Heidelberg Germany; ^3^ Microbial Bioactive Compounds Interfaculty Institute of Microbiology and Infection Medicine University of Tübingen 72076 Tübingen Germany; ^4^ Institute of Molecular Infection Biology University of Würzburg 97080 Würzburg Germany; ^5^ NMR Spectroscopy Analysis Unit German Cancer Research Center (DKFZ) 69120 Heidelberg Germany; ^6^ Department of Pharmaceutical and Bioorganic Chemistry Institute of Pharmacy and Molecular Biotechnology Heidelberg University 69120 Heidelberg Germany; ^7^ Medical Microbiology Interfaculty Institute of Microbiology and Infection Medicine University of Tübingen 72076 Tübingen Germany; ^8^ Department of Infectious Diseases Medical Microbiology and Hygiene Heidelberg University Hospital 69120 Heidelberg Germany

**Keywords:** enterococci, polycationic peptides, resistance, β‐lactam antibiotics

## Abstract

Antibiotic‐resistant enterococci represent a significant global health challenge. Unfortunately, most β‐lactam antibiotics are not applicable for enterococcal infections due to intrinsic resistance. To extend their antimicrobial spectrum, polycationic peptides are conjugated to examples from each of the four classes of β‐lactam antibiotics. Remarkably, the β‐lactam–peptide conjugates gained an up to 1000‐fold increase in antimicrobial activity against vancomycin‐susceptible and vancomycin‐resistant enterococci. Even against β‐lactam‐resistant Gram‐negative strains, the conjugates are found to be effective despite their size exceeding the exclusion volume of porins. The extraordinary gain of activity can be explained by an altered mode of killing. Of note, the conjugates showed a concentration‐dependent activity in contrast to the parent β‐lactam antibiotics that exhibited a time‐dependent mode of action. In comparison to the parent β‐lactams, the conjugates showed altered affinities to the penicillin‐binding proteins. Furthermore, it is found that peptide conjugation also resulted in a different elimination route of the compounds when administered to rodents. In mice systemically infected with vancomycin‐resistant enterococci, treatment with a β‐lactam–peptide conjugate reduced bacterial burden in the liver compared to its originator. Therefore, peptide modification of β–lactam antibiotics represents a promising platform strategy to broaden their efficacy spectrum, particularly against enterococci.

## Graphical abstract

Novel potent compounds are urgently required for the treatment of antibiotic‐resistant bacteria. Conjugation of polycationic peptides to β‐lactam antibiotics extends their efficacy spectrum against enterococci. The β‐lactam–peptide conjugates show significant differences in mechanism and biodistribution compared to their originators. Due to their high potency, the β‐lactam–peptide conjugates can serve as an alternative treatment option for severe enterococcal infections.

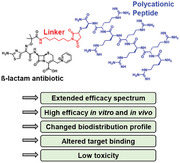

## 1 Introduction

Enterococci are one of the major causes of nosocomial infections associated with high morbidity and mortality rates. Resistance to well‐established antibiotics limits the treatment options for enterococcal infections, highlighting the urgent need for new, potent compounds. Due to the intrinsic resistance of enterococci to the majority of β‐lactam antibiotics, therapy with those antimicrobials is challenging.^[^ ^1^ ^]^ Therefore, treatment of enterococcal endocarditis has traditionally relied on combination therapies involving aminoglycosides.^[^ ^2^ ^]^ However, the increasing emergence of enterococci with high‐level aminoglycoside resistance demands alternative strategies, e.g. dual β‐lactam therapies.^[^ ^3^ ^]^ Combinations of β‐lactam antibiotics with the lipopeptide daptomycin were examined for the treatment of vancomycin‐resistant enterococci (VRE).^[^ ^4^ ^]^ Combination therapy encounters many challenges regarding treatment regimens, and retains the risk of antimicrobial resistance when utilizing established antibiotics.^[^ ^5^ ^]^ β‐Lactam antibiotics with an extended efficacy spectrum against enterococci are therefore greatly required.

The mode of action of β‐lactam antibiotics relies on their binding to penicillin‐binding proteins (PBPs), which prevents cross‐linking of peptidoglycan and thus inhibits bacterial cell wall synthesis.^[^ ^6^ ^]^ Covalent binding to PBPs is mediated by the reactive β‐lactam ring, the common core structure of all β‐lactam antibiotics. Fusion of the β‐lactam ring with an additional heterocycle forms a bicyclic ring system, the characteristic feature of the penicillin, cephalosporin and carbapenem β‐lactam antibiotic subclasses.^[^ ^7^ ^]^ Members of the subclass monobactams possess only the β‐lactam ring moiety and lack fusion to another heterocycle. The interplay between the substituents and the configuration of the β‐lactam antibiotic itself determines the antimicrobial spectrum and influences the interaction with their bacterial targets.^[^ ^8^ ^]^

Intrinsic resistance of enterococci to β‐lactam antibiotics is mainly achieved by the expression of a low‐affinity PBP, PBP5, which leads to a dramatically reduced binding of the β‐lactam to its target.^[^ ^1^, ^9^ ^]^ Recently, it was reported that PBPA, another low‐affinity PBP, is involved in resistance against β‐lactam antibiotics.^[^ ^10^ ^]^ In addition, a two‐component signal transduction system and a protein kinase are assumed to especially promote cephalosporin resistance.^[^ ^1^, ^11^ ^]^ Two members of the fifth‐generation of cephalosporins, ceftaroline and ceftobiprole, that exhibit antimicrobial activity against enterococci were originally designed to regain binding affinity to the key resistance determinant PBP2a of methicillin‐resistant *Staphylococcus aureus* (MRSA).^[^ ^12^ ^]^ However, as both cephalosporins still aim for the original target of β‐lactam antibiotics, their intensive clinical use bears a high risk of resistance development. Therefore, β‐lactam antibiotics with modified mechanisms of action involving a second target are highly desirable.

Multitarget approaches to combat resistance against β‐lactam antibiotics were investigated for resistant Gram‐negative bacteria. β‐Lactam resistance in Gram‐negative bacteria is based on multiple mechanisms, including the acquisition of β‐lactamases, porin mutations and overexpression of efflux pumps. To overcome β‐lactamase‐mediated resistance, combinations of β‐lactam antibiotics with β‐lactamase inhibitors, e.g. the combination of ceftazidime with avibactam are applied.^[^ ^13^ ^]^ In addition to the development of new β‐lactamase‐inhibitors to treat β‐lactam‐resistant Gram‐negative bacteria, structural modification of the β‐lactam scaffolds is used to regain antimicrobial activity against those pathogens.^[^ ^14^ ^]^ One example is the siderophore cephalosporin cefiderocol which is modified with a catechol moiety enabling the active uptake by iron transport channels to penetrate the Gram‐negative cell wall.^[^ ^15^ ^]^ The conjugation of antimicrobial peptides to the β‐lactam core resulting in increased membrane permeabilization capability of the derivative represents an alternative strategy to improve antibacterial activity against Gram‐negative strains.^[^ ^16^ ^]^

To overcome antibiotic resistance, we recently developed a strategy which relies on the modification of established cell‐wall targeting antibiotics, e.g. vancomycin (VAN) conjugated with peptides.^[^ ^17^ ^]^ Following this strategy, here we describe peptide modification of β‐lactam antibiotics. The following representatives of the four β‐lactam classes were chosen as model antibiotics: ceftazidime (CTZ, a cephalosporin), amoxicillin (AMO, a penicillin), ertapenem (ERT, a carbapenem) and aztreonam (AZT, a monobactam). Conjugation was mainly performed with cationic peptides as our previous studies showed that they led to the highest antimicrobial activities.^[^ ^17^ ^]^ The β‐lactam–peptide conjugates were found to exhibit an extended efficacy spectrum, particularly against enterococci, with orders of magnitude increased antimicrobial activity. Most importantly, we have shown that the strategy is applicable to all four β‐lactam classes, suggesting a platform technology for cell wall‐addressing antibiotics in general.

## 2 Results

### 2.1 Synthesis of β‐Lactam–Peptide Conjugates

**Figure 1:**
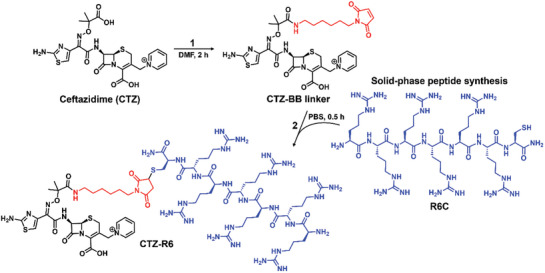
Synthesis of ceftazidime–peptide conjugates. In the first step (1), the BB linker was coupled to the carboxyl group of the methyl propanoic acid moiety of CTZ. Peptides were synthesized by solid‐phase peptide synthesis. In the second step (2), the maleimide functionality of the linker reacts with the thiol group of the cysteine moiety of the peptide sequence (R6C) forming a stable ceftazidime–peptide conjugate (CTZ‐R6).

Peptides were synthesized by solid‐phase peptide synthesis and their identities confirmed by LC/MS (Table S1, Supporting Information). An additional *C*‐terminal cysteine was introduced to all peptides to enable coupling with the maleimide moiety of the respective linker. For the four β‐lactam antibiotic classes, synthesis of the β‐lactam–peptide conjugates was performed in two steps (**Figure** **1**; Figure S1, Supporting Information). In the first step, a heterobifunctional linker was attached to the respective antibiotic, and in the second step, the peptide moiety was conjugated. To avoid interference with the β‐lactams’ pharmacophore antimicrobial properties, the linker coupling position was chosen to be distant from the β‐lactam ring system. Thus, the carboxyl group of the methyl propanoic acid moiety was selected as the linker coupling site for CTZ and AZT whereas for ERT and AMO, the primary amine position was chosen. The intermediate compounds were purified by HPLC, and their identities confirmed by LC/MS (Table S2 and Figures S2–S5, Supporting Information). The linker coupling positions were verified by NMR (Supporting Information). Following conjugation of the intermediates to the peptides, the β‐lactam–peptide conjugates were purified by preparative HPLC, and their identities confirmed by LC/MS (Table S3 and Figures S6–S9, Supporting Information) and NMR (Supporting Information). For designation of the β‐lactam–peptide conjugates, the abbreviation of the β‐lactam antibiotic, following EUCAST standards, combined with a single‐letter code representing the conjugated peptide sequence is used.^[^ ^18^ ^]^ The stability of CTZ‐R6 (structure shown in Figure 1) was evaluated in PBS under various pH conditions using HPLC analysis. CTZ‐R6 was proven to be stable under slightly acidic, basic and neutral pH levels over 24 h (Figure S10, Supporting Information).

### 2.2 Structure–Activity Relationship

**Figure 2:**
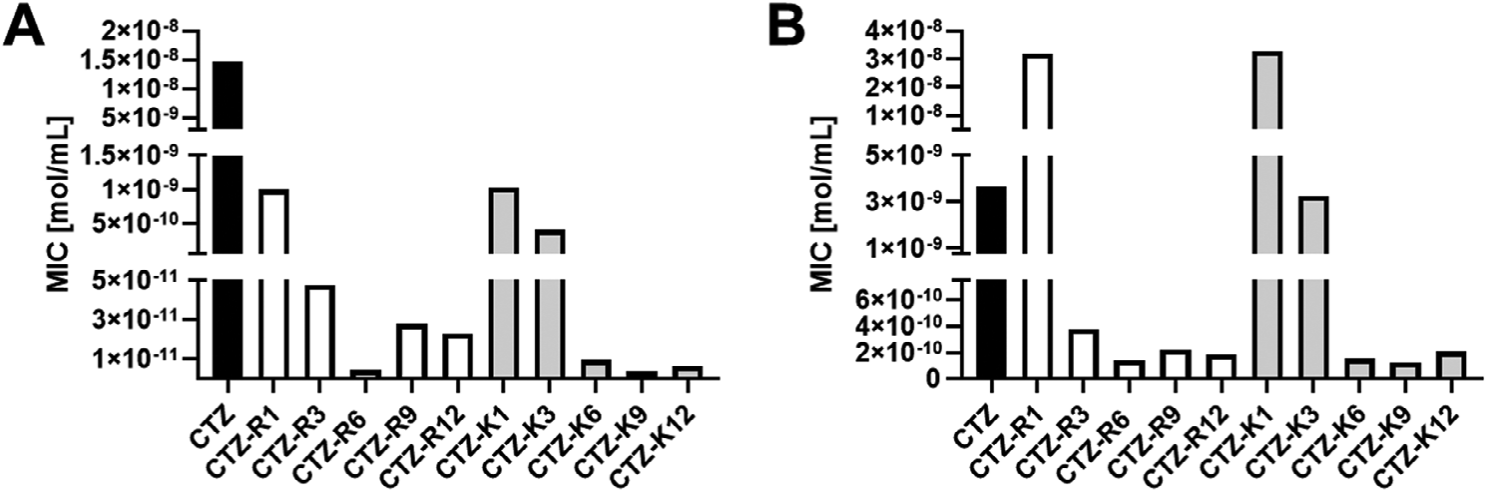
Antimicrobial activity of ceftazidime–peptide conjugates against A) B. subtilis DSM 10 and B) A. bohemicus DSM 100419. With an increasing positive peptide charge in the conjugate, the antimicrobial activity increased independent of the used bacteria. On both bacterial strains, CTZ‐R6 and CTZ‐K9 were the most potent conjugates. Increasing the peptide charge further did not lead to improved antimicrobial activity. Data is shown as median (*n* = 3).

To investigate the influence of the length and charge of the conjugated peptide moiety with respect to the antimicrobial activity of the β‐lactam–peptide conjugates, their MICs were determined and compared to the parent β‐lactams. Here, the comparison of the antimicrobial activity of different CTZ–peptide conjugates is presented against *Bacillus subtilis* and *Acinetobacter bohemicus* as representatives of Gram‐positive and Gram‐negative bacteria, respectively. In general, CTZ–peptide conjugates with cationic peptides showed an increase in antimicrobial activity, whereas conjugates with anionic or neutral charged peptides exhibited decreased or similar activity in comparison to the parent β‐lactam antibiotic (Table S3, Supporting Information). With respect to polycationic peptides, the CTZ–peptide conjugates composed of arginine‐ and lysine‐rich peptides were the most potent ones. With an increasing positive peptide charge, the antimicrobial efficacy of the conjugates increased until reaching maximum effectiveness at a point of optimal peptide charge (**Figure** **2**). For polyarginine containing conjugates, six positive charges were the optimum for antimicrobial activity while for polylysine containing conjugates, the highest antimicrobial activity occurred with nine positive charges. Considering the lower synthesis costs for conjugates with smaller peptides, CTZ coupled to a hexa‐arginine peptide (CTZ‐R6) was chosen as the representative β‐lactam–peptide conjugate for further susceptibility and mode‐of‐action studies. For the AZT–peptide conjugates, a similar correlation between peptide charge and antimicrobial activity was observed (Figure S11, Supporting Information). Therefore, hexa‐arginine conjugates of the other β‐lactam classes, AZT‐R6, ERT‐R6, and AMO‐R6, were used for further investigations.

To prove that conjugation of the peptide to the β‐lactam antibiotic is required for increased antimicrobial activity and the peptide alone does not act as an antimicrobial peptide, the MICs of the different components of CTZ‐R6 were also determined. The BB linker, the peptide and the BB linker coupled to the peptide all showed insignificant activity against different Gram‐positive and Gram‐negative bacteria (**Table** **1** and Table S4, Supporting Information). Furthermore, an equimolar mixture of CTZ and the hexa‐arginine peptide also did not improve antimicrobial activity when compared to CTZ alone (Table S4, Supporting Information). However, the conjugate CTZ‐R6 showed an increased antimicrobial activity against Gram‐positive and Gram‐negative bacteria by a factor of ≈1000 and 2–32, respectively, in comparison to its parent β‐lactam CTZ. As CTZ‐R6 is about three times higher in molecular weight than CTZ, the antimicrobial activity of the conjugate on a molar level is even stronger.

**Table 1:** MICs of the individual components of CTZ‐R6 and the conjugate against different Gram‐positive and CTZ‐sensitive Gram‐negative strains. Data is shown as median (*n* = 3).

	Bacterial strain	MIC [µg mL^−1^]		
		CTZ	BB linker‐R6	CTZ‐R6
Gram‐positive	*B. subtilis* DSM 10	8	>64	0.008
	*E. faecalis* ATCC 29212	4	>64	<0.004
	*E. casseliflavus* ATCC 700327	>2048	>64	0.25
Gram‐negative	*E. coli* Tü20‐025^a)^	2	>64	0.5
	*K. pneumoniae* Tü20‐036^a)^	0.125	>64	0.0625
	*C. freundii* Tü21‐052^a)^	0.25	>64	0.125
	*A. pittii* Tü21‐041^a)^	4	>64	0.125

^a)^ Clinical isolate.

### 2.3 Antimicrobial Spectrum of β‐Lactam–Peptide Conjugates

**Figure 3:**
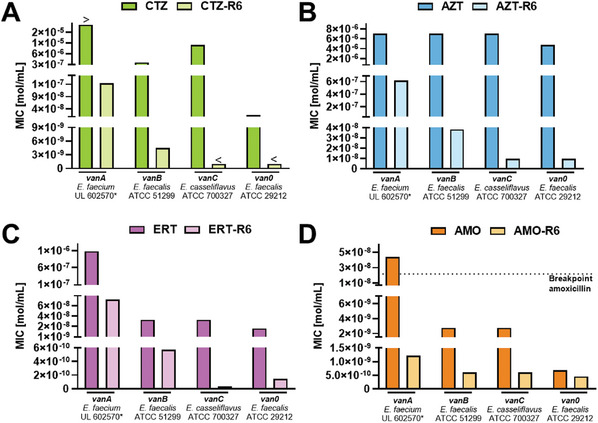
Antimicrobial activity of β‐lactam–peptide conjugates and their parent β‐lactams on enterococci. A) CTZ‐R6, B) AZT‐R6, C) ERT‐R6, and D) AMO‐R6 exhibited superior activity when compared to their parent β‐lactams for several enterococci. Interestingly, with an increasing level of vancomycin resistance the antimicrobial activity of the conjugates decreased. The resistance breakpoint for amoxicillin in enterococci is defined by EUCAST.^[^ ^19^ ^]^ Clinical isolates are highlighted with an asterisk. Data is shown as median (*n* = 3).

To determine the efficacy spectrum of the β‐lactam–peptide conjugates, their antimicrobial activities against different nosocomial pathogens were investigated. Most intriguingly, CTZ‐R6 gained considerable activity against vancomycin‐sensitive and ‐resistant enterococci, showing, for example, about a 300‐fold increased activity against *E. faecalis* (*vanB*) on a molar level (**Figure** **3**A). With a lower level of vancomycin resistance, CTZ‐R6 showed increased antimicrobial activity. A similar activity pattern was observed for the hexa‐arginine conjugates of the three other β‐lactam antibiotics (Figure 3B–D). However, against methicillin‐sensitive and ‐resistant staphylococci, only a slightly improved antimicrobial activity was observed for CTZ‐R6 in comparison to its parent β‐lactam (Table S5, Supporting Information). Synergism between CTZ and CTZ‐R6 on enterococci was not observed (Table S6, Supporting Information). However, combinations of VAN with CTZ or CTZ‐R6 resulted in a similar FIC index <0.5, indicating synergism on *E. faecalis* (Table S6, Supporting Information).

### 2.4 Antimicrobial Spectrum of β‐Lactam–Peptide Conjugates

As shown in Table 1, the antimicrobial activity of CTZ‐R6 against β‐lactam‐sensitive Gram‐negative strains slightly increased in comparison to the parent β‐lactam. For CTZ‐resistant strains, CTZ‐R6 exhibited effects depending on the bacterial species and its antimicrobial resistance properties. CTZ‐R6 exhibited ca. fourfold and 16‐fold increases in antimicrobial activity against Enterobacteriaceae and *A. baumannii* in comparison to CTZ, respectively (**Table** **2**). Against some β‐lactamase‐producing strains, the β‐lactam antibiotics and the respective conjugates were ineffective (**Table** **3**). However, the antimicrobial activity of the derivatives could be restored by the addition of a β‐lactamase inhibitor, as it is well‐known for the parent β‐lactams themselves (Table 3).^[^ ^20^ ^]^ CTZ‐R6 combined with avibactam (AVI), AMO‐R6 with clavulanic acid and AZT‐R6 with AVI were able to prevent hydrolysis of the β‐lactam ring by the β‐lactamases in Gram‐negative strains (Tables S7 and S8, Supporting Information). Antimicrobial activity of the combined conjugate and β‐lactamase inhibitor was slightly increased in comparison to the antimicrobial potency of the parent β‐lactam combined with the same inhibitor.

**Table 2:** MIC ranges of CTZ and CTZ‐R6 on CTZ‐resistant Gram‐negative bacteria. The clinical breakpoints for CTZ are defined by a) EUCAST^[^ ^19^ ^]^ and b) CLSI.^[^ ^21^ ^]^

	Breakpoints CTZ [µg mL^−1^]		MIC range [µg mL^−1^]	
Bacterial species	Sensitive	Resistant	CTZ	CTZ‐R6
*E. coli* (*n* = 3)	≤1^a)^	>4^a)^	32→64	4→64
*K. pneumoniae* (*n* = 4)	≤1^a)^	>4^a)^	64→64	6→64
*C. freundii* (*n* = 3)	≤1^a)^	>4^a)^	32→64	0.5–64
*A. baumannii* (*n* = 4)	≤8^b)^	≥32^b)^	64→64	4–16
*P. aeruginosa* (*n* = 2)	≤0.001^a)^	>8^a)^	32–64	2–16

**Table 3:** Antimicrobial activity of CTZ and CTZ‐R6 alone or in combination with the β‐lactamase inhibitor avibactam on different nosocomial pathogens. In the first column, bacterial strains with their respective β‐lactamases (in brackets) are listed. The addition of AVI restores the activity of CTZ and CTZ‐R6 on strains containing class A (SHV‐18, KPC) and D (Oxa48, Oxa244) β‐lactamases. Hydrolysis of CTZ and CTZ‐R6 by the class D β‐lactamase Oxa23 cannot be prevented by the addition of AVI. Data is shown as median (*n* = 3).

	MIC [µg mL^−1^]				
Bacterial strain	CTZ	CTZ + AVI	CTZ‐R6	CTZ‐R6 + AVI	AVI
*E. coli* ATCC 25 922 (–)	0.25	0.125	0.25	<0.03	16
*E. coli* Tü21‐031^a)^ (Oxa244, ESBL)	32	0.25	4	0.015	–
*K. pneumoniae* ATCC 700 603 (SHV‐18)	64	0.5	6	0.19	>64
*K. pneumoniae* BL809453^a)^ (KPC)	>64	1	>64	0.5	>64
*K. pneumoniae* 0 6841/Nr.45^a)^ (Oxa48)	>64	1	>64	0.5	>64
*A. baumannii* SC300007^a)^ (Oxa23)	>64	>64	>64	>64	>64

^a)^ Clinical isolate.

### 2.5 Mode of Action Studies

**Figure 4:**
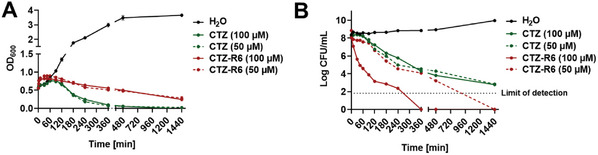
Time‐kill analysis of CTZ‐R6 and CTZ on B. subtilis. A) After addition of CTZ and CTZ‐R6 to an exponentially growing culture, the OD_600_ values constantly decreased independent of the applied concentration. B) Determination of the CFU mL^−1^ revealed a faster killing of CTZ‐R6 when compared to CTZ. CTZ showed a time‐dependent killing mechanism, whereas CTZ‐R6 exhibited a concentration‐dependent mode of action. Data is shown as mean (*n* = 3).

To investigate the killing mechanism of the β‐lactam–peptide conjugates, their time‐kill kinetics were determined using the model organism *B. subtilis*. OD_600_ measurements revealed constantly decreasing turbidity at the applied concentrations for both CTZ and CTZ‐R6 (**Figure** **4**A). For CTZ, a constant decrease in colony‐forming units (CFU) mL^−1^ over 24 h independent of the applied concentration was observed demonstrating a time‐dependent killing mechanism (Figure 4B), as typically known for β‐lactam antibiotics.^[^ ^22^ ^]^ At equimolar concentrations, CTZ‐R6 showed faster killing compared to its parent β‐lactam and 100 µM of CTZ‐R6 reduced the number of CFU to the detection limit after 6 h (6 log units drop). Interestingly, halving the conjugate concentration resulted in a slower killing rate, suggesting a concentration‐dependent killing mechanism for CTZ‐R6. Similar observations were made for the hexa‐arginine conjugates of the other three β‐lactam antibiotics (Figure S12, Supporting Information).

**Figure 5:**
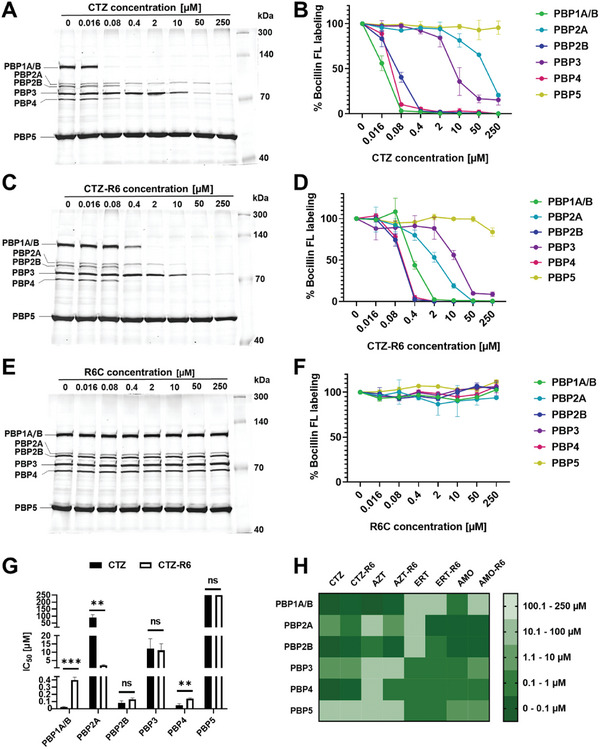
Determination of the PBP binding profile of CTZ, CTZ‐R6, and R6C to B. subtilis PBPs. Representative SDS‐PAGE gel images for inhibition of PBPs by A) CTZ, C) CTZ‐R6, and E) R6C are depicted. Quantitative analysis of gel bands for B) CTZ, D) CTZ‐R6, and F) R6C. Data is shown as means ± SD from three independent experiments. G) Comparison of IC_50_ values for CTZ and CTZ‐R6 for the different PBPs. H) Heatmap giving an overview of the PBP binding profiles for the different β‐lactam–peptide conjugates and their parent β‐lactams.

For investigation of the interaction between the original targets of β‐lactam antibiotics, the PBPs, and the β‐lactam–peptide conjugates, a binding assay with the PBPs in membrane fractions of *B. subtilis* was performed using competition with the fluorescent β‐lactam Bocillin FL penicillin as readout. The PBP inhibition profile for CTZ suggested a strong binding of CTZ to PBP1A/B, PBP2B, and PBP4 (**Figure** **5**A,B). While CTZ‐R6 showed affinity for the same PBPs, PBP2A was targeted in addition (Figure 5C,D). For the hexa‐arginine peptide alone, binding to the PBPs of *B. subtilis* was not observed at the concentrations tested (Figure 5E,F). Interestingly, the IC_50_ values for the competition assay suggest that binding to PBP1A/B and PBP4 by CTZ‐R6 was decreased in comparison to CTZ, whereas affinity to PBP2A was significantly increased (Figure 5G). Similar to CTZ‐R6, the three other hexa‐arginine β‐lactam–peptide conjugates also exhibited significant differences in their PBP binding profile in comparison to their parent β‐lactams (Figure S13, Supporting Information). As observed for CTZ‐R6, AZT‐R6, and ERT‐R6 showed a higher affinity to PBP2A in comparison to their parent β‐lactams (Figure 5H). The assay was also performed with PBPs isolated from vancomycin‐resistant *E. faecalis* to determine if the β‐lactam–peptide conjugates could attain affinity to the low‐affinity PBP5 causing intrinsic resistance to β‐lactam antibiotics. For CTZ‐R6 and AZT‐R6, a slightly improved binding to PBP5 was observed, however, at considerably higher concentrations than their MICs (Figures S14 and S15, Supporting Information). Additionally, for CTZ‐R6 the spontaneous resistance rate is very low (< 2.7 × 10^−9^).

### 2.6 Evaluation of Cytotoxicity and Pharmacokinetics

**Figure 6:**
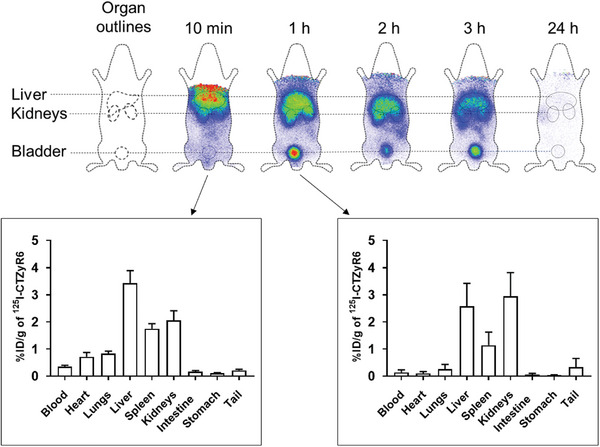
Scintigraphic images and biodistribution of CTZ‐R6 in Wistar rats. The β‐lactam–peptide conjugate shows up to 3 h post injection a high accumulation in the liver and in the kidneys by scintigraphy. No signal from ^125^I‐CTZ‐yR6 could be detected 24 h post injection. Biodistribution studies 10 min and 1 h post injection confirmed the high accumulation in the liver and in the kidneys. Data is shown as mean + SD (*n* = 3).

The viability of HEK‐293 and HEPG2 cells after incubation with CTZ and CTZ‐R6 was investigated. For these cell lines, no significant cytotoxicity up to a concentration of 512 µg mL^−1^ of CTZ or CTZ‐R6 was observed (Figure S16, Supporting Information). In addition, for all β‐lactam–hexa‐arginine conjugates, the absence of hemolytic effects was also demonstrated (Figure S17, Supporting Information). To study the biodistribution of the β‐lactam–peptide conjugates, scintigraphical images in mice and rats were recorded. Within this study, no adverse effects related to the administration of our compound were observed. For radiolabeling purposes, the peptide sequence of the conjugates was elongated by an additional d‐tyrosine (Table S3, Supporting Information). As for most β‐lactam antibiotics,^[^ ^23^ ^]^ CTZ‐K6 exhibited renal elimination (Figure S18, Supporting Information) whereas CTZ‐R6 accumulated in the liver and in the kidneys (**Figure** **6**). The rapid liver accumulation was also observed for the hexa‐arginine conjugates of the three other β‐lactam classes (Figure S19, Supporting Information). Biodistribution studies of CTZ‐R6 confirmed accumulation in the liver, and to a lesser extent, in the spleen and in the kidneys 10 min post injection, while after 1 h a similar amount of CTZ‐R6 was found in the liver and in the kidneys (Figure 6).

### 2.7 In Vivo Efficacy in a Systemic Infection VRE Mouse Model

**Figure 7:**
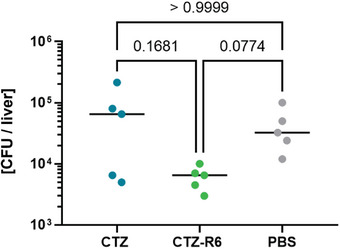
Bacterial burden in the liver of VRE infected mice after CTZ‐R6 treatment. Treatment with CTZ‐R6 resulted in a decreased bacterial number of VRE (vancomycin‐ and ceftazidime‐resistant *E. faecium* UW18830) in the liver of infected female Balb/c mice at 48 h p.i. Shown are individual values per mouse (*n* = 5), the median per group and the p‐values of the Kruskal‐Wallis test.

To determine the therapeutic efficacy of CTZ‐R6 in comparison to its originator CTZ in vivo, a systemic VRE infection in mice was performed and the mice were treated with the respective compounds. Within this study, no adverse effects related to the administration of our compound were observed. The CFU in the liver were determined to demonstrate the therapeutic efficacy after treatment, as CTZ‐R6 accumulates mainly in the liver (as shown in biodistribution studies, Figure 6). Treatment of VRE‐infected mice with CTZ‐R6 reduced the bacterial burden in the liver when compared to mice treated with CTZ or PBS (**Figure** **7**).

## 3 Discussion

The derivatization of β‐lactam antibiotics with polycationic peptides resulted in highly potent conjugates with increased antimicrobial activity against Gram‐positive bacteria, e.g., enterococci, when compared to their parent β‐lactams. Antimicrobial activity of the β‐lactam–peptide conjugates was influenced by the charge and amino acid composition of the conjugated peptide. With an increasing peptide charge, the antimicrobial activity of the respective conjugates increased continuously until reaching an optimal value beyond which further increase in charge did not lead to significantly improved antimicrobial activity. Among the β‐lactam–polyarginine conjugates, derivatives with six arginines exhibited the highest antimicrobial activity, which is in concordance with the findings of Umstätter et al. regarding vancomycin conjugates.^[^ ^17^ ^]^ Within the group of β‐lactam–polylysine conjugates, derivatives with nine lysine moieties were the most potent compounds. Although lysine and arginine have the same net charge, arginine residues with their guanidinium groups can potentially form more hydrogen bonds with membrane bilayers than lysine.^[^ ^23^, ^24^ ^]^ This can possibly result in a stronger and prolonged interaction with the bacterial cell wall and thus increased antimicrobial activity for the β‐lactam–hexa‐arginine conjugate.

Enterococci are known to be intrinsically resistant to the majority of β‐lactam antibiotics.^[^ ^25^ ^]^ The conjugation of cationic peptides to β‐lactam antibiotics regained antimicrobial activity against different enterococcal strains. These findings were evident for all four classes of β‐lactam antibiotics suggesting a platform technology for cell‐wall targeting antibiotics in general. Remarkably, peptide conjugates of the monobactam AZT, which is known to exhibit no antimicrobial activity against Gram‐positive bacteria in general, showed high efficacy against *B. subtilis* and enterococci.^[^ ^26^ ^]^ However, the differences in antimicrobial activity of the β‐lactam–hexa‐arginine conjugates against enterococci with different resistance types were observed. Their antimicrobial potency against *E. faecalis* (*vanB*) and *E. casseliflavus* (*vanC*) was significantly higher in comparison to *E. faecium* (*vanA*). Despite being rarely reported for *E. faecium* strains, the lower antimicrobial activity could be caused by the occurrence of β‐lactamases.^[^ ^27^ ^]^ The antimicrobial activity of the β‐lactam–peptide conjugates against Gram‐negative bacteria was at least preserved, or could be restored, by the addition of a β‐lactamase inhibitor. These results are even more remarkable when considering that the β‐lactam–peptide conjugates are up to threefold higher in molecular weight than their corresponding parent β‐lactam. Normally, the porin size restricts the entry of such large molecules into the Gram‐negative cell wall, thereby restricting their antimicrobial activity.^[^ ^28^ ^]^ Taken together, the β‐lactam–peptide conjugates showed a broadening of the efficacy spectrum when compared to their respective parent β‐lactam antibiotics. To our knowledge, no peptide modification approach to increase antimicrobial activity against Gram‐positive bacteria addressing all subclasses of β‐lactam antibiotics has been reported so far.

In microdilution studies, we could show that the hexa‐arginine peptide alone and as an equimolar mixture with a β‐lactam antibiotic exhibited no significant antimicrobial activity. These findings imply that covalent linkage between the peptide and the β‐lactam is required for the increased antimicrobial activity. This leads to the assumption that the hexa‐arginine moiety could function as a cell‐penetrating vehicle enabling the β‐lactam antibiotic to be conveyed as cargo into the bacterial periplasm of Gram‐negative cells. However, as the β‐lactam–hexa‐arginine conjugates showed also increased activity against Gram‐positive bacteria, its site of action cannot be restricted to the outer membrane. Further studies are ongoing to clarify the molecular sites and modes of action of this class of new compounds. Using checkerboard studies, we observed synergism between VAN and CTZ‐R6 against vancomycin‐resistant enterococci in a manner similar to VAN combined with CTZ. Synergism between vancomycin and β‐lactam antibiotics on enterococci has been described previously.^[^ ^29^ ^]^ Peptide conjugation to β‐lactam antibiotics does not appear to alter the synergistic effects. Fortunately, no cytotoxic effects using two human cell lines were determined and no lysis was observed affirming the bacteria‐specific activity of the β‐lactam–peptide conjugates. Performing a preliminary pharmacodynamic exploration, we revealed that the β‐lactam–peptide conjugates exhibited concentration‐dependent rather than time‐dependent killing, the latter being demonstrated for their parent β‐lactams. This dramatic change in the kill kinetics would necessitate the adaptation of the treatment scheme but would nevertheless enable new therapy options. With a PBP binding assay, we could show that the PBP binding profile of the β‐lactam–peptide conjugates in *B. subtilis* differs from the parent β‐lactams. However, no increased binding affinity toward the low‐affinity PBP5 of *E. faecalis*, the determinant of intrinsic resistance to β‐lactam antibiotics, was observed. The hexa‐arginine peptide alone also showed no binding to PBPs. These results suggest that the β‐lactam–peptide conjugates act, in addition to their binding to PBPs, with a second mechanism of action.

In line with the vancomycin conjugate FU002, the β‐lactam–hexa‐arginine conjugates showed an altered biodistribution profile in comparison to their parent β‐lactams.^[^ ^17^ ^]^ While β‐lactam antibiotics are known to be eliminated primarily through the kidneys, the β‐lactam–hexa‐arginine conjugates exhibited a high accumulation in both the liver and in the kidneys.^[^ ^30^ ^]^ Therefore, the pharmacokinetics of the conjugates could be potentially favorable for the treatment of patients with renal impairment. Most importantly, we could demonstrate that CTZ‐R6 showed therapeutic efficacy in a murine VRE infection model when compared to its originator CTZ. This finding suggests an alternative VRE therapeutic approach using the β‐lactam–peptide conjugates described herein. In subsequent studies, the correlation between applied dose and resulting efficacy should be thoroughly investigated.

## 4 Conclusion

Conjugation of polycationic peptides to β‐lactam antibiotics resulted in broadening of their efficacy spectrum. Due to their high potency in vitro and in vivo, the β‐lactam–peptide conjugates could serve as a treatment option for enterococcal infections. We revealed major differences in their mechanism and biodistribution compared to their parent β‐lactams. Therefore, further studies are required to completely understand the relationship between the structure of the β‐lactam–peptide conjugates and their mode of action.

## 5 Experimental Section

### Materials

Fmoc‐l‐amino acids were purchased from Orpegen Peptide Chemicals GmbH, Heidelberg, Germany. The rink amide resin, Fmoc(fluorenylmethoxycarbonyl)‐d‐tyrosine, dimethyl formamide (DMF) and 2‐(1*H*‐benzotriazol‐1‐yl)‐1,1,3,3‐tetramethyluronium hexafluorophosphate (HBTU) were supplied by Iris Biotech GmbH, Marktredwitz, Germany. *N*,*N*‐diisopropylethylamine (DIPEA), piperidine and trifluoroacetic acid (TFA) were obtained from Biosolve BV, Valkenswaard, The Netherlands. Dichloromethane (DCM) was purchased from Fisher Scientific GmbH, Schwerte, Germany. Gradient grade acetonitrile for HPLC was provided by WICOM Germany GmbH, Heppenheim, Germany. Dulbecco phosphate buffered saline (PBS), acetic acid, diethyl ether, dimethyl sulfoxide (DMSO), methanol, HPLC grade water, triisopropyl silane (TIS), Triton X‐100, 3,6‐dioxa‐1,8‐octanedithiol (DODT), *N*,*N*,*N*′,*N*′‐tetramethyl‐*O*‐(*N*‐succinimidyl)uronium tetrafluoroborate (TSTU), cation‐adjusted Mueller Hinton Broth II (MHB II) medium and amoxicillin were purchased from Sigma‐Aldrich Chemie GmbH, Taufkirchen, Germany. Ceftazidime was purchased from Dr. Friedrich Eberth Arzneimittel, Ursensollen, Germany. Aztreonam and potassium clavulanate were supplied by Cayman Chemical, Ann Arbor, MI, USA, ertapenem by InfectoPharm und Consilium GmbH, Heppenheim, Germany and sodium avibactam by Hycultec GmbH, Beutelsbach, Germany. Sulfosuccinimidyl‐4‐(*N*‐maleimidomethyl)cyclohexane‐1‐carboxylate (Sulfo‐SMCC) was obtained from Carbosynth Limited, Compton, Berkshire, UK. BD Bacto Agar was supplied by Becton Dickinson, Franklin Lakes, NJ, USA. A Densicheck plus densitometer was obtained from bioMerieux Deutschland GmbH, Nürtingen, Germany and 96‐well microtiter plates (polypropylene, u‐bottom and polystyrene, v‐ and flat‐bottom) from Greiner Bio‐One International GmbH, Kremsmünster, Austria. Bacterial strains and clinical isolates were provided by the Institute for Medical Microbiology and Hygiene, Heidelberg University Hospital, Heidelberg, Germany and by the Interfaculty Institute for Medical Microbiology and Hygiene, University of Tübingen, Tübingen, Germany. Fetal bovine serum was purchased from Lonza Verviers SPRL, Verviers, Belgium, Gibco, Trypan blue solution (0.4%) and Gibco Trypsin‐EDTA (0.25%) from Life Technologies GmbH, Darmstadt, Germany, Costar Corning 96‐well plates from Corning Inc., Tewksbury, MA, USA and PrestoBlue from Fisher Scientific, Schwerte, Germany. Iodine‐125 was obtained from Hartmann Analytic GmbH, Braunschweig, Germany. Wistar rats (RjHan:WI), Swiss mice (RjOrl:SWISS) and Balb/c mice (BALB/cJRj) were supplied by Janvier Labs, Le Genest‐Saint‐Isle, France.

### Compound Analysis and Purification

Analytical reversed‐phase HPLC was performed using a C18 column (Chromolith Performance RP‐18e, 100 × 3 mm; Merck KGaA, Darmstadt, Germany) coupled to an Agilent 1100 series system (Agilent Technologies Inc., Santa Clara, USA) with UV detection at 214 nm. Compounds were separated using a linear gradient from water (+ 0.1% TFA) to acetonitrile (+ 0.1% TFA) over 5 min at a flow rate of 2 mL min^−1^. Peptides, intermediates and β‐lactam–peptide conjugates were purified by preparative reversed‐phase HPLC using a Reprosil Pur 120 C18‐AQ (5 µm, 250 × 25 mm) column (Dr. Maisch HPLC GmbH, Ammerbuch, Germany) coupled to a Gilson 331 series system (Gilson Inc., Middleton, WI, USA) with UV detection at 214 nm. If not stated otherwise, peptides and reaction products were purified using a linear gradient from water (+ 0.1% TFA) to acetonitrile (+ 0.1% TFA) over 15 min at a flow rate of 20 mL min^−1^. The purity and identity of the compounds were determined by LC/MS using a Thermo Fisher Exactive Orbitrap MS system (Thermo Fisher Scientific Inc., Waltham, MA, USA). After purification, the compounds were lyophilized using an Alpha 2–4 LD plus system (Martin Christ GmbH, Osterode, Deutschland).

### Peptide Synthesis

Peptides were synthesized by Fmoc solid‐phase peptide synthesis. A rink amide resin (loading 0.59 mmol g^−1^; 0.15 mmol synthesis scale) was weighed into a syringe containing a porous filter plate and swelled by incubating with DCM for 15 min. The Fmoc protecting group of the resin was cleaved by incubating the resin twice with 20% piperidine in DMF for 5 min, after which the resin was washed thrice with DMF. The Fmoc‐protected amino acid (4 eq), HBTU (3.8 eq), and DIPEA (8 eq, 5.5 M) were dissolved in DMF and the resulting solution drawn up into the syringe containing the deprotected resin. After shaking at room temperature for 1 h, the resin was washed thrice with DMF. Deprotection, washing, and coupling steps were repeated until the desired number of amino acids had been coupled. For the synthesis of polyarginine peptides, a double coupling instead of a single coupling strategy was performed. After completion of the peptide synthesis, the resin was washed with DMF, DCM, and diethyl ether (three times each) followed by drying under vacuum. The peptide was cleaved from the resin by incubation with an aqueous solution of TFA (95%) and TIS (2.5%) for 2 h. Peptides with arginine moieties were cleaved using an aqueous solution of TFA (92.5%), TIS (2.5%) and DODT (2.5%). After incubation, the cleaved peptide was precipitated by diethyl ether and the precipitate centrifuged at 4000 rpm for 5 min. The resulting pellet was dried under vacuum. The peptides were purified and analyzed by LC/MS as described above.

### Synthesis of BB Linker

The 6‐maleinimidohexanamide linker (hereafter, BB linker) was synthesized in three reaction steps. First, *N*‐Boc‐1,6‐diaminohexane (4.62 mmol, 1 eq) and maleic anhydride (4.43 mmol, 1 eq) were dissolved in DCM and incubated on ice for 1 h. The solution was then washed with water and saturated aqueous sodium chloride followed by drying over sodium sulfate. After filtering off the sodium sulfate, the solution was concentrated down. Next, sodium acetate (2.87 mmol, 1 eq) was added to the resulting amide solution and the mixture heated in an oil bath at 120 °C. After 3 h, the reaction mixture was cooled in an ice bath and saturated aqueous sodium hydrogen carbonate was added. The mixture was then extracted with DCM and the organic phase was washed, dried and concentrated as described above. The concentrate was dissolved in MeOH and purified by preparative HPLC as described above. Finally, the *tert*‐butyloxycarbonyl (Boc) protecting group was cleaved using 30% TFA in DCM while shaking for 30 min. The product was washed with acetonitrile, concentrated and lyophilized.

### Synthesis of BB Linker

For the synthesis of the CTZ–BB linker intermediate, ceftazidime (0.05 mmol, 1 eq) and TSTU (0.05 mmol, 1 eq) were dissolved in DMF followed by the addition of DIPEA (0.059 mmol, 1.2 eq). After 15 min shaking at room temperature, the BB linker (0.06 mmol, 1.2 eq) dissolved in DMF and DIPEA (0.059 mmol, 1.2 eq) was added to the reaction mixture. Following 1.5 h shaking at room temperature, the solvent was evaporated, and the concentrate dissolved in a 1:1 mixture of water and acetonitrile. The CTZ–BB linker intermediate was purified by preparative HPLC and subsequently lyophilized. Synthesis of the AZT–BB linker intermediate followed the same experimental protocol.

### Synthesis of AMO–SMCC and ERT–SMCC Intermediates

For the synthesis of the AMO–SMCC intermediate, amoxicillin (0.05 mmol, 1 eq) dissolved in DMSO and the crosslinker Sulfo‐SMCC (0.05 mmol, 1 eq) dissolved in water and DIPEA (0.05 mmol, 1 eq) were mixed and shaken at room temperature for 2 h. Afterwards, the AMO–SMCC intermediate was purified by preparative HPLC using 0.1% CH_3_COOH instead of 0.1% TFA as an eluent additive to maintain stability of the bicyclic β‐lactam ring system. Synthesis of the ERT–SMCC intermediate followed the same experimental protocol except that ertapenem (0.04 mmol, 1 eq) and Sulfo‐SMCC (0.02 mmol, 0.5 eq) were dissolved in water.

### Peptide Conjugation to β‐Lactam Antibiotics

Conjugation of various peptide moieties to the β‐lactam linker intermediates was performed in the same manner for all four β‐lactam antibiotics. Briefly, 1 eq of the β‐lactam linker intermediate (CTZ–BB linker, AZT–BB linker, AMO–SMCC and ERT–SMCC) and 2 eq of the peptide were dissolved in PBS at pH = 5.4 and mixed. If the pH value of the reaction mixture dropped below 5.4, it was readjusted with DIPEA. After 30 min shaking at room temperature, the β‐lactam–peptide conjugate was purified by preparative HPLC (for amoxicillin and ertapenem conjugates, 0.1% CH_3_COOH was added to the eluent) and lyophilized as described above.

### NMR

NMR spectra were acquired using a Bruker Avance Neo (Bruker Corporation, Billerica, MA, USA) operating at 700 and 176 MHz for ^1^H and ^13^C nuclei, respectively, and a Varian VNMRS (Varian Inc., Palo Alto, CA, USA) operating at 500 and 125 MHz for ^1^H and ^13^C nuclei, respectively, in *d*_6_‐DMSO at 25 °C. The chemical shifts of ^1^H and ^13^C nuclei are reported relative to the solvent signals (*δ*_H,d5‐DMSO_ = 2.50 ppm and *δ*_C,d6‐DMSO_ = 39.52 ppm).

### Stability Assessment of CTZ‐R6

To determine the stability of CTZ‐R6, the conjugate was dissolved in PBS (pH 5.4/7.4/9.4; adjusted with HCl or NaOH) at a concentration of 1 mg mL^−1^. The solution was incubated on a shaker at 700 rpm and room temperature for 24 h. Samples were taken after 0 min, 10 min, 30 min, 1 h, 2 h, 4 h, 6 h, and 24 h and the remaining content of CTZ‐R6 was subsequently analyzed by RP‐HPLC at 214 nm.

### Stability Assessment of CTZ‐R6

The minimum inhibitory concentration (MIC) of the β‐lactam–peptide conjugates was determined by broth microdilution as previously described.^[^ ^17^ ^]^ Briefly, in cation‐adjusted MHB II medium, a twofold serial dilution of the compounds was prepared in 96‐well round‐bottom polystyrene or polypropylene microtiter plates with each well containing 50 µL. The plates were inoculated with 50 µL bacterial suspension and the final inoculum, in a total volume of 100 µL, contained 5 × 10^5^ CFU mL^−1^. The microplates were incubated for 20 h at 37 ± 1 °C. The lowest concentration preventing visible bacterial growth was considered the MIC value. For susceptibility testing of combinations of β‐lactamase‐inhibitors with either a β‐lactam antibiotic or a β‐lactam–peptide conjugate, fixed concentrations of the inhibitors according to the EUCAST (European Committee on Antimicrobial Susceptibility Testing) guidelines (clavulanic acid = 2 mg L^−1^, avibactam = 4 mg L^−1^) were used.

### Checkerboard Assay

Synergies between ceftazidime and CTZ‐R6, ceftazidime and vancomycin (VAN), and CTZ‐R6 and VAN were investigated on the vancomycin‐resistant enterococci *E. faecalis* ATCC 51 299 and *E. faecium* UL 602 570. The assay was performed as described by Bellio et al.^[^ ^31^ ^]^ Briefly, the antibiotic stock solutions in 0.9% NaCl were serially diluted in cation‐adjusted MHB II in separate 96‐well plates. Next, 25 µL of each antibiotic dilution was transferred horizontally and vertically to the final plate. The preparation and addition of the bacterial inoculum and plate incubation were performed as described in the section “Determination of the minimum inhibitory concentration”. Interactions between the antibiotics were calculated using the fractional inhibitory concentration (FIC) index.

(1) $$FIC\;index=\frac{MIC_{AB}}{MIC_{A}}+\frac{MIC_{BA}}{MIC_{B}}$$

MIC_A_ = MIC of antibiotic A; MIC_B_ = MIC of antibiotic B, MIC_AB_ = MIC of antibiotic A when used in combination with antibiotic B; MIC_BA_ = MIC of antibiotic B when used in combination with antibiotic A.

Synergism was defined as a FIC index ≤ 0.5, indifference as a FIC index between > 0.5 and 4 and antagonism as a FIC index > 4.^[^ ^32^ ^]^

### Time‐Kill Assay

For determination of time‐kill kinetics of the β‐lactam–peptide conjugates and their parent β‐lactams, 14 mL of cation‐adjusted MHB II medium was inoculated with an *B. subtilis* DSM 10 overnight culture to obtain an OD_600_ of 0.5, corresponding to ≈10^8^ CFU mL^−1^. To this exponentially growing culture, 50 or 100 µm of each of the antibiotics was added except for a negative control culture which was treated with only water. The bacterial cultures were incubated at 30 °C and 200 rpm and the OD_600_ was measured on 1 mL aliquots taken at 0, 10, 30, 45, 60, 90, 120, 240, 360, 480 and 1440 min. Additionally, at the same time points, 15 µL of serial tenfold dilutions of the bacterial suspension were plated onto MHB II agar plates. The agar plates were incubated overnight at 30 °C. The next day, bacterial colonies were counted and the CFU mL^−1^ were calculated (limit of detection: 10^1.8^ CFU mL^−1^). For each antibiotic the assay was performed in triplicate (three biological replicates).

### Determination of Spontaneous Resistance Rate

The rate of spontaneous resistance of *E. casseliflavus* (ATCC 700 327) against CTZ‐R6 was investigated as previously described.^[^ ^33^ ^]^ A bacterial suspension of ATCC 700 327 was adjusted to an OD_600_ of 1 with 0.9% NaCl, corresponding to a theoretical concentration of 2 × 10^8^ CFU mL^−1^. 250 µL of the suspension were plated on 10 cation‐adjusted MHB II agar plates containing 16 µg mL^−1^ CTZ‐R6 and incubated at 37 °C for 18 h. As concentration control, the bacterial inoculum (OD_600_ = 1) was diluted and plated on antibiotic‐free cation adjusted MHB II agar plates and incubated at 37 °C for 18 h. The number of colonies on both types of plates was counted and the spontaneous resistance rate was calculated accordingly:

(2) $$\begin{array}{ccc} & & Spontaneous\;Resistance\;Rate\\ & & =\frac{\#\;colonies\;on\;antibiotic\;plates}{\#\;colonies\;on\;control\;plate\;x\;dilution\;factor\;x\;2.5} \end{array}$$

### Extraction of Membrane‐Bound Proteins

*B. subtilis* DSM 10 and *E. faecalis* ATCC 51 299 (equal to DSM 12 956) membrane protein fractions were extracted according to the protocol of Dean et al. with minor modifications.^[^ ^34^ ^]^ From a growing overnight bacterial culture, 3 L of LB (Luria‐Bertani) medium or TSB (tryptic soy broth) were inoculated with *B. subtilis* DSM 10 and *E. faecalis* ATCC 51 299, respectively, to obtain an OD_600_ = 0.3. The cultures were grown to an OD_600_ = 0.6–0.8 while shaking at 37 °C and cells were harvested by centrifugation at 4000 g for 30 min at 4 °C. The resulting pellet was washed twice with pre‐cooled PBS and resuspended in 30 mL of ice‐cooled PBS. The suspension was sonicated on ice (10 cycles of 30 s at 50% power for *B. subtilis*; 12 cycles of 30 s at 80% power for *E. faecalis;* 2 min intervals between cycles) using an Omni Ruptor 4000 ultrasonic homogenizer (Omni International, Kennesaw, GA, USA). Afterwards, the suspension was centrifuged at 7000 g for 10 min at 4 °C. The clear supernatant was transferred into a 50 mL polypropylene tube and centrifuged in a Beckman Coulter centrifuge equipped with a JA‐20 fixed‐angle rotor (Beckman Coulter, Krefeld, Germany) at 50 000 g for 1 h at 4 °C. The resulting pellet was washed with ice‐cold PBS and centrifuged a second time. The pellet was resuspended in 2 mL PBS and aliquots were stored at −80 °C. The protein content was determined using Bradford reagent (VWR International, Darmstadt, Germany) and Pierce BCA (bicinchoninic acid) protein assay kits (Thermo Fisher Scientific, Waltham, MA, USA) for membrane fractions of *B. subtilis* and *E. faecalis*, respectively.

### Gel‐Based PBP Affinity Assay

For determination of PBP affinity, the parent β‐lactam or the respective conjugate was serially diluted in PBS to obtain concentrations ranging from 0.016–250 µM. Each dilution was combined with *B. subtilis* (30 µg) or *E. faecalis* (15 µg) extracted membrane fractions. As reference samples, membrane fractions incubated with only PBS were prepared. Reaction mixtures were incubated for 20 min at 37 °C while shaking at 400 rpm. Next, Bocillin FL penicillin (Thermo Fisher Scientific, Waltham, MA, USA) was added to obtain a final concentration of 20 µM in the mixture. Samples were incubated for 30 min at 37 °C while shaking at 400 rpm. To denature the samples, 4× Laemmli sample buffer (Bio‐Rad Laboratories, Feldkirchen, Germany) was added, and the samples incubated for 10 min at 70 °C. Afterwards, the samples were centrifuged for 5 min at 13 000 rpm. Samples were separated by SDS‐PAGE using a 10% Tris‐glycine gel for 100 min at 180 V. The bands were visualized using a Typhoon FLA 9500 imager (GE Healthcare, Chicago, IL, USA) with excitation at 473 nm. The gel images were analyzed using ImageJ software. Brightness and contrast were adjusted to improve the signal‐to‐noise ratio and the background signal was subtracted over the whole image. Next, integrated density values of the gel bands were measured and Bocillin FL penicillin labeling of each PBP in antibiotic‐treated samples were set in proportion to the reference sample. IC_50_ values were calculated with GraphPad Prism software (version 8.0.1) using inhibitor versus normalized response variable slope and least‐squares fit. If GraphPad Prism determined an ambiguous number and inhibition was not seen at 250 µM, the IC_50_ was assigned to > 250 µM. The mean was calculated using the IC_50_ values of at least two independent assays for each antibiotic.

### Hemolysis Assay

The hemolytic activities of the β‐lactam–peptide conjugates were determined by incubating the compounds with purified human erythrocytes from three different male donors. For each β‐lactam, the hemolysis assay was performed in duplicate and was repeated three times. The compounds were dissolved in PBS (12.8 mg mL^−1^) and were twofold serially diluted in a 96‐well plate (polystyrene, v‐bottom) resulting in a volume of 50 µL per well. 10% Triton X‐100 in PBS and PBS only were used as positive and negative controls, respectively. To every well, 50 µL of purified erythrocytes were added to obtain final concentrations of the conjugates ranging from 1.25–640 µg mL^−1^. The microplate was incubated for 60 min at 37 °C. Afterwards, 75 µL PBS was added to each well and the plate centrifuged for 5 min at 2000 rpm. 50 µL aliquots of the supernatant from each well were then transferred to a fresh 96‐well plate (polystyrene, flat‐bottom) and the absorption measured using a Tecan Infinite M200 PRO microplate reader (Tecan, Männedorf, Switzerland) at 554 nm. For the calculation of the hemolytic activity of the β‐lactam–peptide conjugates, the following equation was used:

(3) $$Hemolysis\;\;\left[\%\right]=100\;x\;\frac{A_{Substance}-A_{Blank}}{A_{Triton}-A_{Blank}}\;\;\;A\;\text{…}\;Absorption$$

### Cytotoxicity Assay

To exclude any cytotoxic effects on mammalian cells prior to in vivo studies, the viability of the cell lines HEP‐G2 (hepatocellular carcinoma) and HEK‐293 (human primary embryonal kidney) after incubation with different concentrations of the β‐lactam–peptide conjugates were determined. For the viability assay, 10^4^ cells per well were seeded in a 96‐well half area microplate and incubated overnight in a humidified 5% CO_2_ atmosphere at 37 °C. The next day, the conjugates were twofold serially diluted in PBS and added to the cells to obtain final concentrations ranging from 1–512 µg mL^−1^. Cells were then incubated with the antibiotics in a humidified 5% CO_2_ atmosphere at 37 °C for a further 24 h. PrestoBlue HS Cell Viability Reagent was then added to the wells and the plates again were incubated in a humidified 5% CO_2_ atmosphere at 37 °C for 1 h. Afterwards, the absorption at 570 nm and 600 nm (reference) using an Infinite M200 PRO microplate reader (Tecan, Männedorf, Switzerland) was measured. Cell viability was calculated using the untreated control cells as a reference for 100% alive cells.

### In Vivo Systemic Infection Mouse Model of Vancomycin‐Resistant *Enterococcus Faecium*

The animal infection studies were approved by the local government of Lower Franconia, Germany (approval number 55.2.2‐2532.2‐1283) and performed in strict accordance with the guidelines for animal care and experimentation of the German Animal Protection Law and the DIRECTIVE 2010/63/EU of the EU. The animals were housed in individually ventilated cages under standardized lighting conditions and had ad libitum access to food and water. To evaluate the in vivo activity during systemic infection, female Balb/c mice were infected with 5×10^7^ CFU of VRE *E. faecium* UW 18 830 (*vanA*) by tail vein injection. Beginning at 1 h p.i., then repeatedly every 12 h, mice received intravenously either CTZ (30 mg kg body weight^−1^ day^−1^), CTZ‐R6 (30 mg kg body weight^−1^ day^−1^) or sterile PBS. The livers were recovered 48 h p.i. (12 h after the last treatment) and frozen at – 80 °C until further processing. For the determination of the bacterial burden, the livers were thawed, homogenized in PBS and plated in serial dilutions on brain heart infusion agar plates. CFU were counted after overnight incubation at 37 °C.

### Radiolabeling

To enable radiolabeling of the β‐lactam–peptide conjugates, their peptide sequence was elongated by an N‐terminal d‐tyrosine. A 1 mm stock solution of the respective compound (CTZ‐yR6, CTZ‐yK6, AZT‐yR6, AMO‐yR6, and ERT‐yR6) in 0.25 m phosphate buffer (pH = 7.5) was prepared. The solution was mixed with 5 MBq of iodine‐125 and radiolabeling was performed using the chloramine T method as previously described.^[^ ^35^ ^]^ After 30 s, the reaction was quenched by the addition of a saturated methionine solution and the radiolabeled compound purified by reversed‐phase preparative radio‐HPLC (1100 series, Agilent Technologies, Santa Clara, CA, USA). Analysis was performed by analytical radio‐HPLC (1100 series, Agilent Technologies, Santa Clara, CA, USA) using a linear gradient from water (+ 0.1% TFA) to acetonitrile (+ 0.1% TFA) over 5 min at a flow rate of 2 mL min^−1^.

### Scintigraphy

All animal studies were approved by the Animal Care and Use committees from Regierungspräsidium Karlsruhe (Karlsruhe, Germany; reference number 35–9185.81/G‐199/21; date of approval: November 23, 2021). To obtain scintigraphic images, 1–3 MBq of the respective ^125^I‐radiolabeled β‐lactam–peptide conjugate was intravenously injected into the tail vein of isoflurane anaesthetized female Wistar rats or Swiss mice. Scintigraphic images were taken with a γ‐camera (Biospace Lab, Nesles‐la‐Vallée, France) at 10 min, 1, 2, 3, and 24 h after injection of the compounds.

### Biodistribution

The biodistribution of CTZ‐yR6 in Wistar rats was determined at 10 min and 3 h post injection. Rats were euthanized at the defined time points and organs (heart, liver, spleen, kidney, intestine, and stomach), blood samples, and tail were removed and weighed. Using a Cobra γ‐counter (Packard Bioscience, Meriden, CT, USA), the amount of radioactivity in each sample was measured which was then used in combination with the organ weight and injected dose for the calculation of the total injected dose per gram of tissue (%ID g^−1^).

### Statistical Analysis

Statistical analysis was performed using GraphPad Prism (version 8.0.1). For comparison of two groups, statistical analysis was performed using unpaired t‐test and for more than three groups, one‐way ANOVA (n.s. p > 0.05, *p < 0.05, **p < 0.01, ***p < 0.001). For the systemic infection mouse model, the Kruskal‐Wallis test was applied.

## Conflict of Interest

The authors declare no conflict of interest.

## Supporting information



Supporting Information

## Data Availability

The data that support the findings of this study are available from the corresponding author upon reasonable request.
