# Supplementary material for: Conjugation of Polycationic Peptides Extends the Efficacy Spectrum of β‐Lactam Antibiotics
Source: Adv Sci (Weinh). 2024 Nov 5;11(48):2411406. doi: 10.1002/advs.202411406 (PMC11672299; doi:10.1002/advs.202411406)
Supplement: Supplementary file 1 — Supporting Information [file ADVS-11-2411406-s001.docx]

Supporting Information

**Conjugation of polycationic peptides extends the efficacy spectrum of β-lactam antibiotics**

*Julia Werner, Florian Umstätter, Manuel B. Böhmann, Hannah Müller, Barbro Beijer, Tobias Hertlein, Laura Kaschnitz, Veronika Bram, Christian Kleist, Karel D. Klika, Silke Peter, Eric Mühlberg, Gabriel Braune, Sabrina Wohlfart, Martin Gärtner, Stefan Zimmermann, Uwe Haberkorn, Knut Ohlsen, Heike Brötz-Oesterhelt, Walter Mier and Philipp Uhl**

**Table of Contents**

1. Peptides prepared by solid-phase peptide synthesis II

2. Synthesis scheme for the β-lactam–peptide conjugates III

3. LC/MS analysis of intermediate compounds III

4. NMR analysis VII

5. LC/MS analysis of the β-lactam–peptide conjugates and their MICs on *B. subtilis* DSM10 XI

6. Stability assessment of CTZ-R6 XV

7. Antimicrobial activities of AZT–peptide conjugates XVI

8. Antimicrobial activities of individual components of CTZ-R6 XVI

9. Antimicrobial activities of β-lactam–peptide conjugates against staphylococci XVI

10. Testing of synergism XVII

11. Antimicrobial activities of β-lactam–peptide conjugates and β-lactam inhibitor combinations against Gram-negative bacteria XVIII

12. Time-kill studies XIX

13. PBP binding profiles XX

14. Cytotoxicity evaluation XXII

15. Pharmacokinetics XXIV

16. References XXV

# **1. Peptides prepared by solid-phase peptide synthesis**

**Table S1.** Peptides prepared by solid-phase peptide synthesis. The table lists the peptide sequences in single-letter code with their corresponding calculated molecular ions, calculated charged ions and observed ions.

| **Peptide sequence** | **Calculated molecular ion [Da]** | **Calculated charged ion [*m*/*z*, Da]** | **Observed ion [*m*/*z*, Da]** |
| --- | --- | --- | --- |
| R1C | 277.12 | 277.12 | 277.14 [M − H + H^+^]^+^ |
| R3C | 589.32 | 589.32 | 589.33 [M − H + H^+^]^+^ |
| R6C | 1057.63 | 529.32 | 529.32 [M − H + 2H^+^]^2+^ |
| R9C | 1525.93 | 509.32 | 509.31 [M − H + 3H^+^]^3+^ |
| R12C | 1994.23 | 665.75 | 665.75 [M + 3H^+^]^3+^ |
| K1C | 249.11 | 249.11 | 249.14 [M − H + H^+^]^+^ |
| K3C | 505.30 | 506.31 | 506.31 [M + H^+^]^+^ |
| K6C | 889.59 | 445.30 | 445.30 [M − H + 2H^+^]^2+^ |
| K9C | 1273.87 | 1273.87 | 1273.88 [M + H^+^]^+^ |
| K12C | 1658.16 | 829.58 | 829.59 [M − H + 2H^+^]^2+^ |
| (GR)_3_C | 760.39 | 760.39 | 760.40 [M + H^+^]^+^ |
| R3K3C | 973.61 | 973.61 | 973.62 [M + H^+^]^+^ |
| (KR)_3_C | 973.61 | 487.31 | 487.32 [M − H + 2H^+^]^2+^ |
| E6C | 895.28 | 895.28 | 895.28 [M + H^+^]^+^ |
| G6C | 463.15 | 463.15 | 463.17 [M + H^+^]^+^ |
| yK6C | 1052.65 | 526.83 | 526.83 [M − H + 2H^+^]^2+^ |
| yR6C | 1220.70 | 610.85 | 610.86 [M − H + 2H^+^]^2+^ |

**2. Synthesis scheme for the β-lactam–peptide conjugates**

**Figure S1.** Synthesis scheme for the (A) AZT–, (B) ERT– and (C) AMO–peptide conjugates. In the first step (1), a linker is coupled to the β-lactam antibiotic. In the second step (2), the peptide moiety is conjugated to the intermediate resulting in the final β-lactam–peptide conjugate. For designation of the β-lactam–peptide conjugates, the abbreviation of the β-lactam antibiotic, following EUCAST standards, combined with a single-letter code representing the conjugated peptide sequence is used.^[1]^


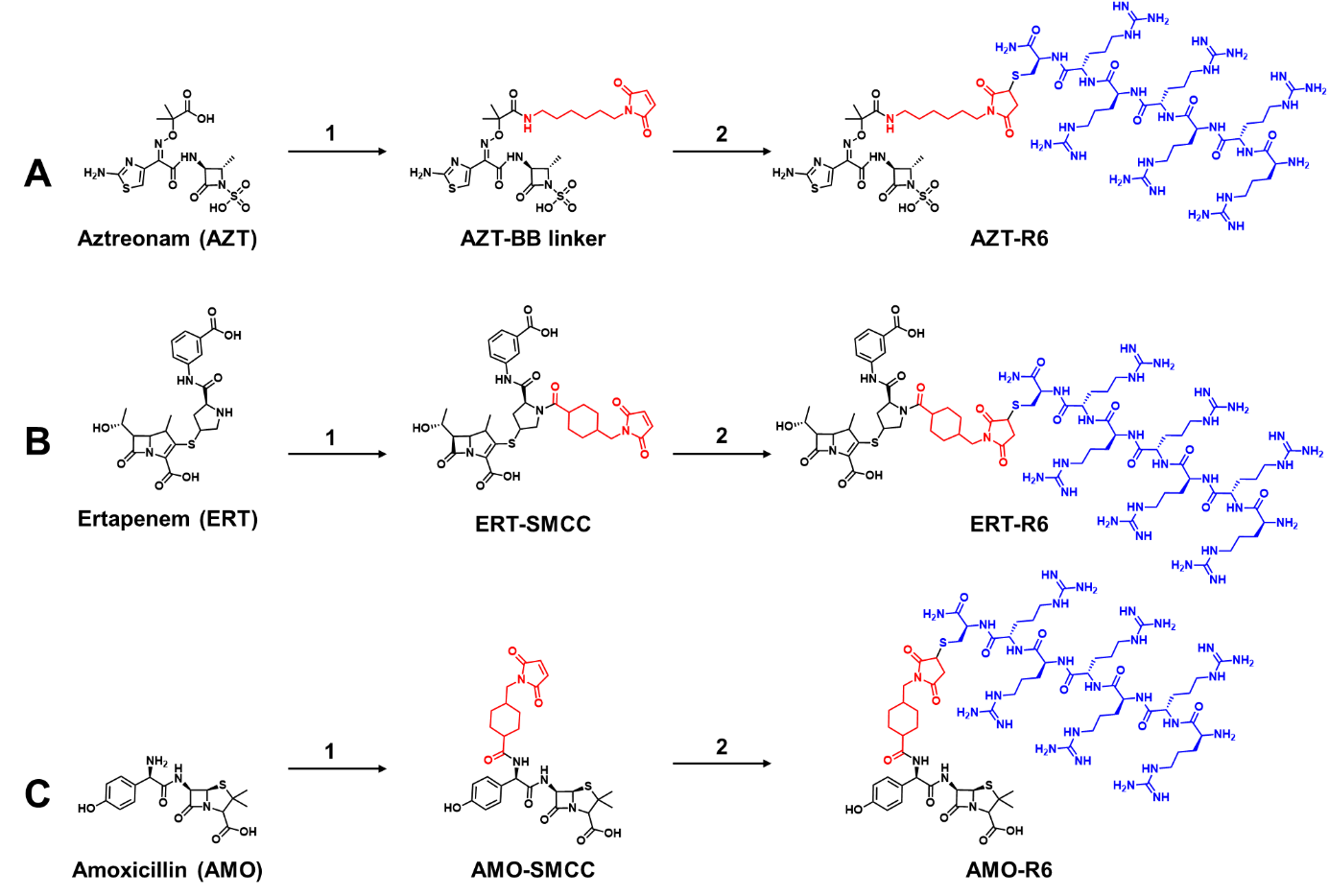


# **3. LC/MS analysis of intermediate compounds**

## **3.1. List of intermediate compounds**

**Table S2**. Synthesized BB linker and β-lactam–linker intermediates. The table lists the synthesized BB linker and β-lactam–linker intermediates with their corresponding calculated molecular ions, calculated charged ions and observed ions.

| **Intermediate** | **Calculated molecular ion [Da]** | **Calculated charged ion [*m*/*z*, Da]** | **Observed ion [*m*/*z*, Da]** |
| --- | --- | --- | --- |
| BB linker | 196.12 | 197.13 | 197.13 [M + H^+^]^+^ |
| CTZ–BB linker | 724.21 | 725.22 | 725.21 [M + H^+^]^+^ |
| AZT–BB linker | 613.16 | 614.17 | 614.17 [M + H^+^]^+^ |
| AMO–SMCC | 584.19 | 585.20 | 585.20 [M + H^+^]^+^ |
| ERT–SMCC | 694.23 | 695.24 | 695.23 [M + H^+^]^+^ |
| BB linker–R6C | 1253.75 | 627.38 | 627.38 [M − H + 2H^+^]^2+^ |

## **3.2 HPLC profile and mass spectrum of CTZ–BB linker**

**Figure S2.** HPLC profile, mass spectrum and chemical structure of CTZ–BB linker. Assignments: 725.21 [M + H^+^]^+^, 646.17 [M − py + H^+^]^+^.


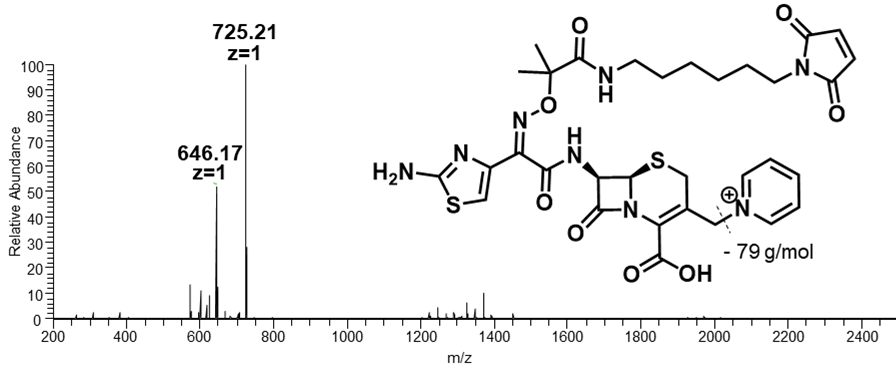

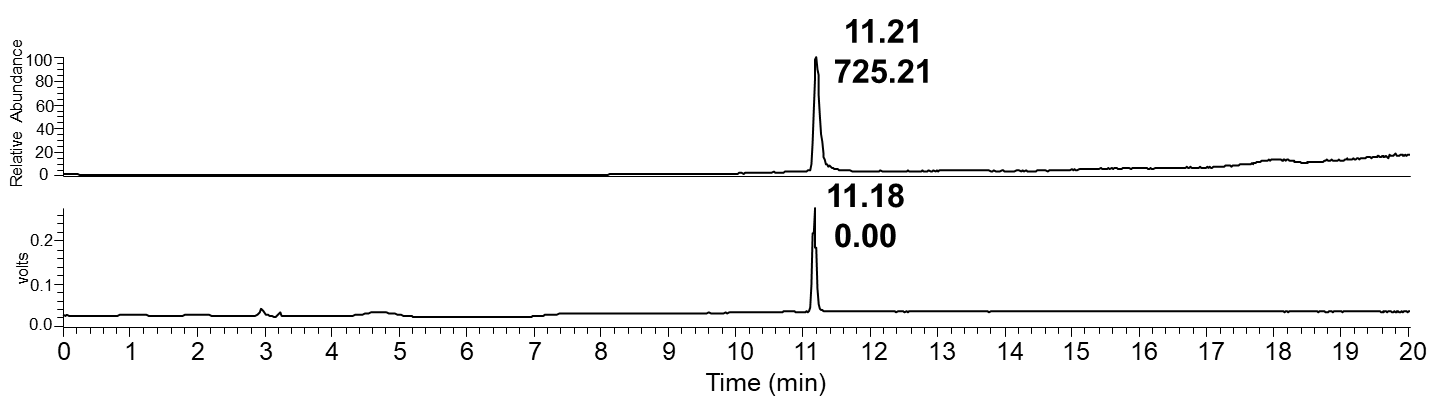


## **3.3. HPLC profile and mass spectrum of AZT–BB linker**

**Figure S3.** HPLC profile, mass spectrum and chemical structure of AZT–BB linker. Assignments: 614.17 [M + H^+^]^+^, 534.21 [M − SO_3_ + H^+^]^+^.


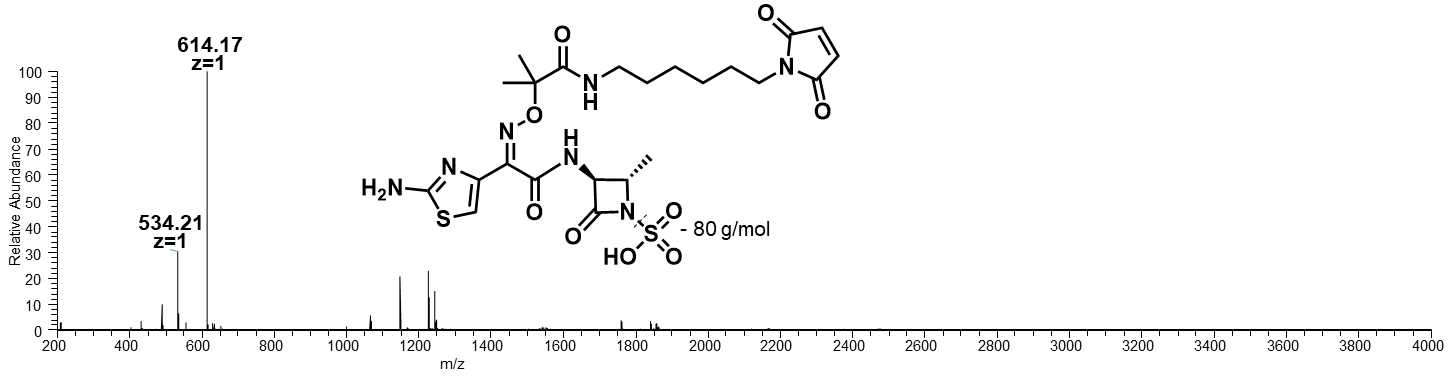

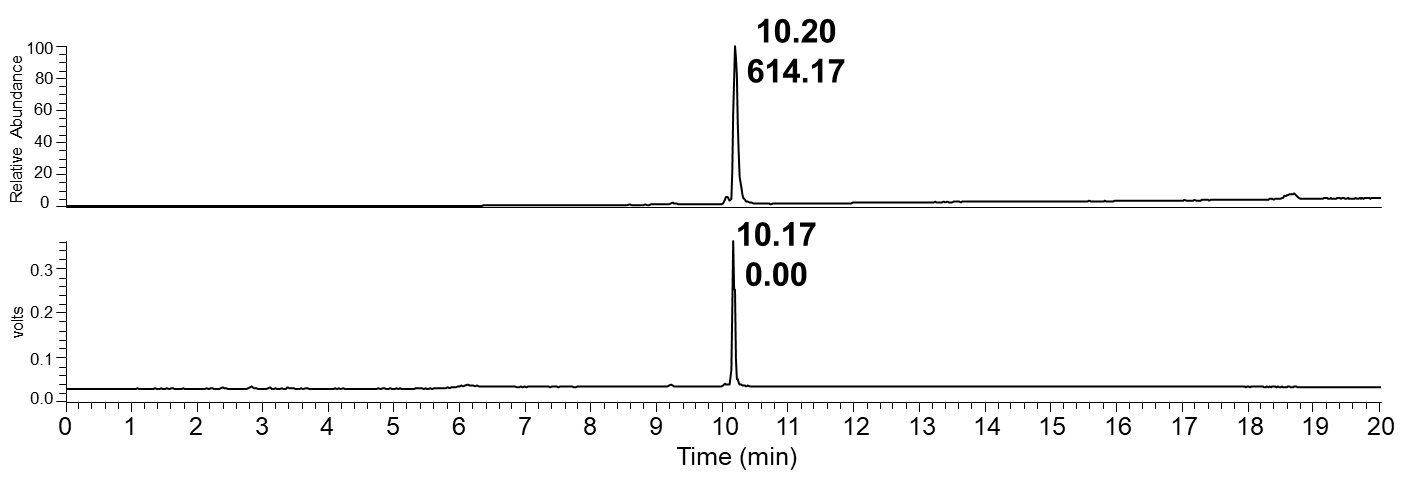


## **
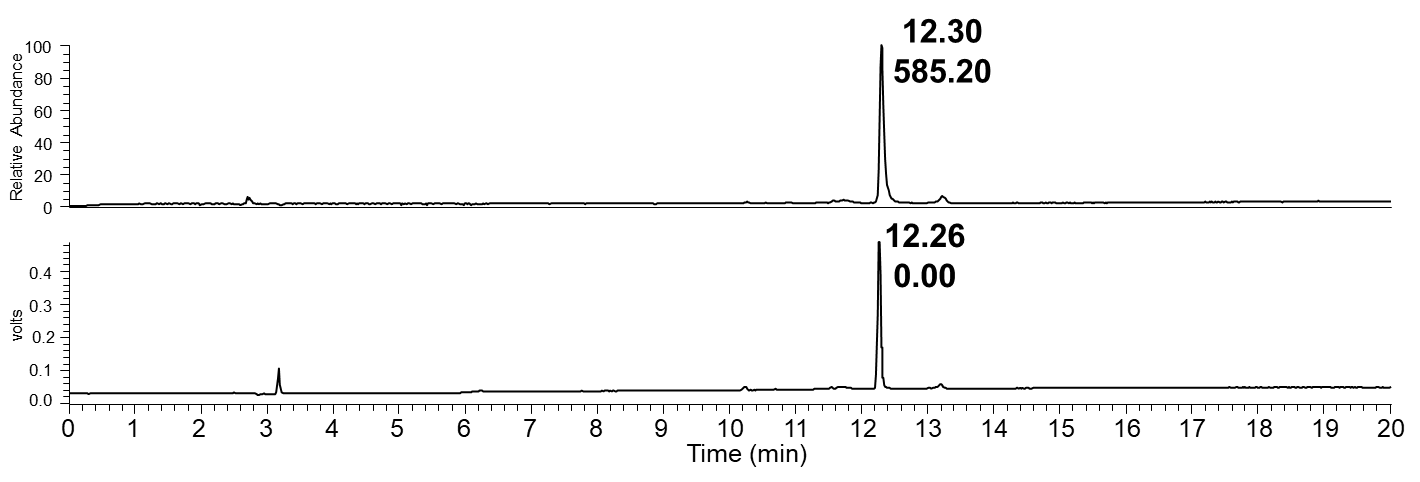
**
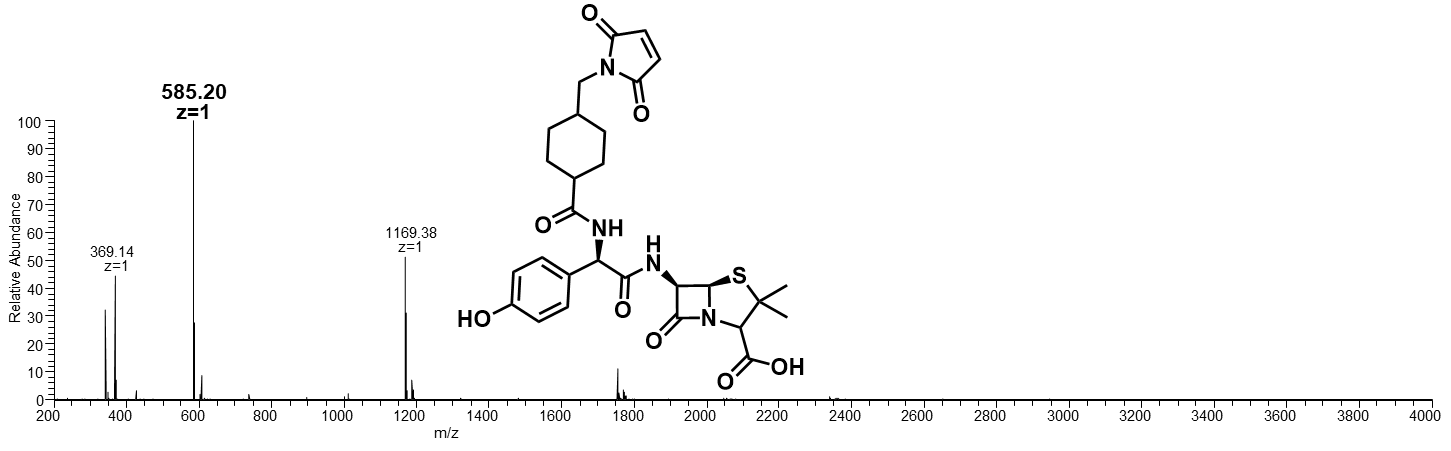
**3.4. HPLC profile and mass spectrum of AMO–SMCC**

**Figure S4.** HPLC profile, mass spectrum and chemical structure of AMO–SMCC. Assignments: 585.20 [M + H^+^]^+^, 1169.38 [2M + H^+^]^+^.

##
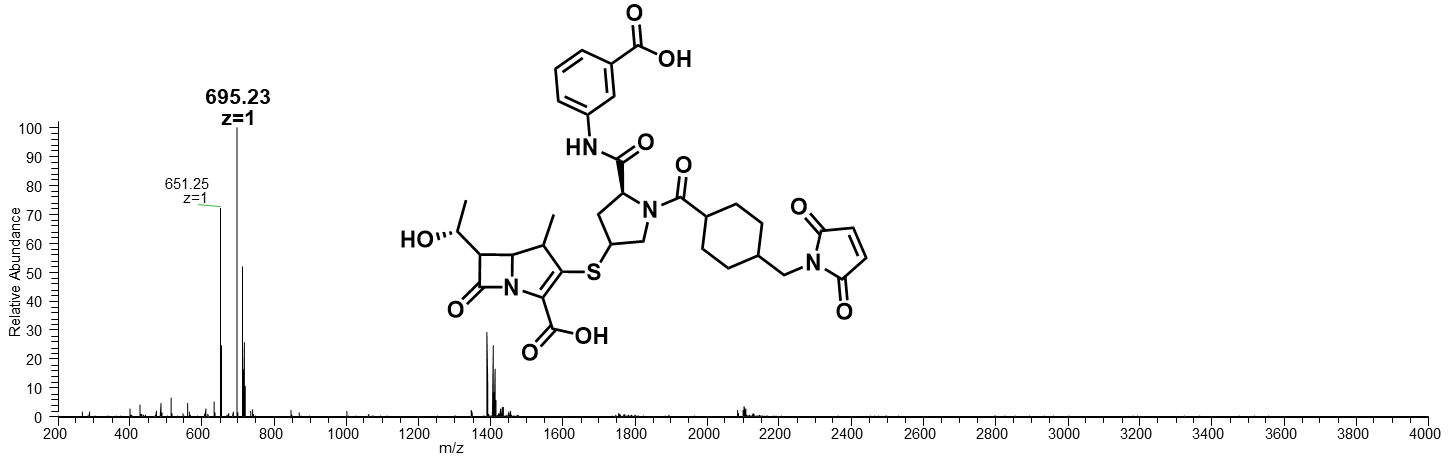

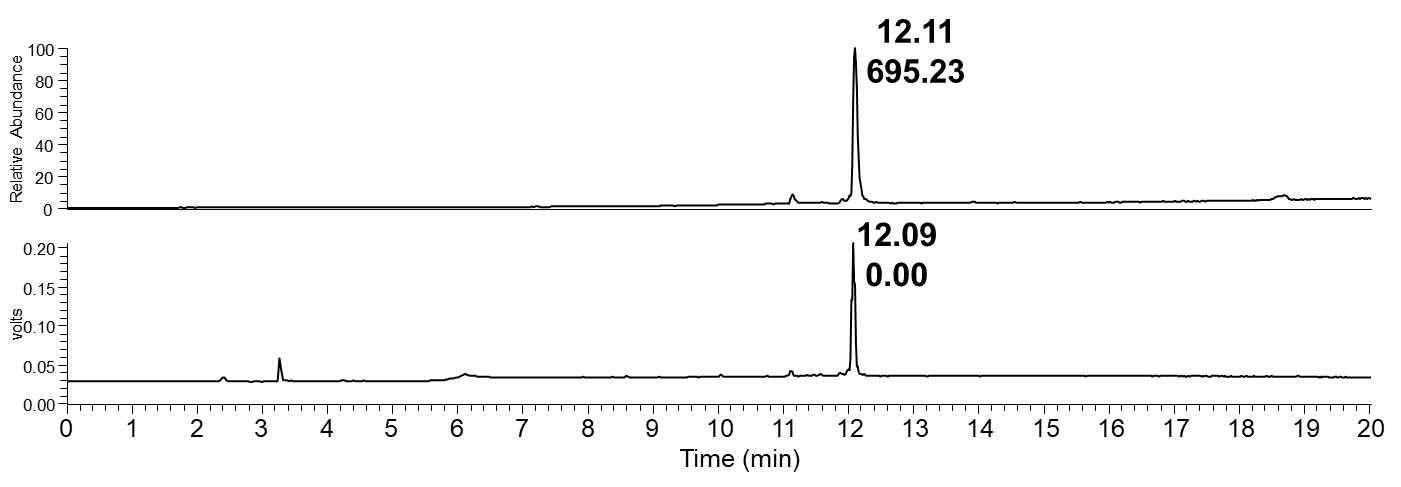
**3.5. HPLC profile and mass spectrum of ERT–SMCC**

**Figure S5.** HPLC profile, mass spectrum and chemical structure of ERT–SMCC. Assignments: 695.23 [M + H^+^]^+^, 651.25 [M − CO_2_ + H^+^]^+^.

**4. NMR analysis**

## **4.1 BB linker**

^1^H NMR (700 MHz, d_6_-DMSO): *δ* = 1.19–1.24 (m, 2H), 1.27–1.32 (m, 2H), 1.45–1.52 (ol m, 4H), 2.753 (ho m, 2H), 3.386 (t, 2H, *J* = 7.12 Hz), 7.005 (s, 2H), 7.745 (br s, 3H). ^13^C NMR (176 MHz, d_6_-DMSO): *δ* = 25.27 (t), 25.64 (t), 26.84 (t), 27.80 (t), 36.95 (t), 38.70 (t), 134.49 (d), 171.13 (s).

## **4.2. CTZ**

^1^H NMR (500 MHz, d_6_-DMSO): *δ* = 1.36 (s, 3H), 1.40 (s, 3H), 3.06 (d, 1H, *J* = 17.6 Hz), 3.49 (d, 1H, *J* = 17.8 Hz), 5.05 (d, 1H, *J* = 5.1 Hz), 5.18 (d, 1H, *J* = 13.5 Hz), 5.66 (d, 1H, *J* = 13.4 Hz), 5.73 (dd, 1H, *J* = 8.2, 5.0 Hz), 6.69 (s, 1H), 7.20 (s, 2H), 8.13 (t, 2H, *J* = 6.8 Hz), 8.56 (tt, 1H, *J* = 7.7, 1.4 Hz), 9.45 (d, 2H, *J* = 5.8 Hz), 10.85–11.35 (br s, 1H). ^13^C NMR (125 MHz, d_6_-DMSO): *δ* = 24.16 (qt), 24.67 (qt), 24.79 (t), 57.48 (d), 58.50 (d), 61.54 (t), 83.05 (s), 108.87 (s), 109.45 (d), 128.02 (d), 138.60 (s), 143.20 (s), 145.12 (d), 145.55 (d), 149.82 (s), 162.85 (s), 163.03 (s), 163.67 (s), 168.24 (s), 176.17 (s).

**4.3. CTZ–BB linker**

^1^H NMR (500 MHz, d_6_-DMSO): *δ* = 1.05–1.18 (m, 4H), 1.26–1.42 (m, 10H), 2.94–3.07 (m, 3H), 3.29–3.39 (m, 2H), 3.53 (d, 1H, *J* = 17.6 Hz), 5.10 (d, 1H, *J* = 5.0 Hz), 5.13 (d, 1H, *J* = 13.0 Hz), 5.68 (d, 1H, *J* = 13.3 Hz), 5.73 (dd, 1H, *J* = 8.1, 4.9 Hz), 6.75 (s, 1H), 7.00 (s, 2H), 7.12 (t, 1H, *J* = 5.9 Hz), 7.27 (s, 2H), 8.16 (t, 2H, *J* = 6.9 Hz), 8.59 (t, 1H, *J* = 7.7 Hz), 9.49 (d, 2H, *J* = 5.8 Hz), 9.63 (d, 1H, *J* = 8.1 Hz). ^13^C NMR (125 MHz, d_6_-DMSO): *δ* = 24.09 (qt), 24.55 (t), 24.68 (qt), 25.62 (t), 25.78 (t), 27.85 (t), 28.86 (t), 36.98 (t), 38.34 (t), 57.44 (d), 58.50 (d), 61.61 (t), 83.20 (s), 108.32 (s), 109.54 (d), 127.94 (d), 134.43 (d), 138.95 (s), 142.49 (s), 145.27 (d), 145.59 (d), 149.91 (s), 162.20 (s), 162.42 (s), 163.22 (s), 168.50 (s), 171.10 (s), 173.28 (s).

**4.4. CTZ–R6 (minor species indicated with appended “m”)**

^1^H NMR (500 MHz, d_6_-DMSO): *δ* = 1.10–1.21 (ol m), 1.25 (d, *J* = 6.5 Hz), 1.27 (d, *J* = 6.7 Hz), 1.29–1.44 (ol m), 1.37 (d, *J* = 5.1 Hz), 1.41m (d, *J* = 5.3 Hz), 1.44–1.62 (ol m), 1.62–1.75 (ol m), 2.29m (t, *J* = 7.4 Hz), 2.44–2.49 (ol m), 2.75 (s), 2.87 (dd, *J* = 12.9 and 8.6 Hz), 2.98 (t, *J* = 7.1 Hz), 3.00–3.27 (ol m), 3.28–3.36 (ol m), 3.40 (br d_AB_, *J* = −18.3 Hz), 3.49 (s), 3.50m (s), 3.55 (br d_AB_, *J* = −18.4 Hz), 3.59–3.66 (ol m), 3.84 (br qt, *J* = 5.4 Hz), 4.02 (ddd, *J* = 3.9, 8.7, and −15.4 Hz), 4.19–4.30 (ol m), 4.34 (br qt, *J* = 5.5 Hz), 4.38 (qn, *J* = 7.1 Hz), 5.13m (s), 5.23 (d, *J* = 5.0 Hz), 5.25m (d, *J* = 3.9 Hz), 5.46 (s), 5.51m (s), 5.54 (s), 5.58–5.62 (ol m), 5.63m (s), 5.94 (dd, *J* = 8.0 and 4.9 Hz), 6.78 (s), 6.87m (s), 7.06m (br s), 7,12–7.17 (ol m), 7.26 (br s), 7.52m (br s), 7.57m (br s), 7.74–7.88 (ol m), 7.93 (br qt, *J* = 5.5 Hz), 8.09 (br t, *J* = 8.6 Hz), 8.12–8.28 (ol m), 8.62 (m), 8.65 (tt, *J* = 7.8 and 1.3 Hz), 9.01m (d, *J* = 5.8 Hz), 9.07 (d, *J* = 5.9 Hz), 9.74 (d, *J* = 7.9 Hz), 9.75m (d, *J* = 8.0 Hz). ^13^C NMR (125 MHz, d_6_-DMSO): *δ* = 1.2 (qt), 12.3 (qt), 16.7 (qt), 18.0 (qt), 24.1 (t), 24.23 (qt), 24.24 (qt), 24.5 (qt), 24.6 (qt), 24.98 (t), 25.0 (t), 25.1 (t), 25.27 (t), 25.32 (t), 25.5 (t), 25.7 (t), 25.86 (t), 25.88 (t), 26.0 (t), 27.0 (t), 28.5 (t), 28.9 (t), 29.10 (t), 29.17 (t), 29.2 (t), 29.3 (t), 32.8 (t), 33.1 (t), 33.4 (t), 35.0 (t), 35.7 (t), 35.8 (t), 37.8 (t), 38.2 (t), 38.5 (t), 40.2 (t), 40.5 (t), 41.8 (t), 50.6 (d), 51.8 (d), 52.0 (d), 52.25 (d), 52.30 (d), 52.48 (d), 52.55 (d), 52.6 (d), 53.0 (d), 53.6 (d), 57.6 (d), 59.0 (d), 60.2 (d), 60.8 (t), 63.9 (t), 69.5 (t), 69.9 (t), 83.3 (t), 83.4 (t), 109.78 (d), 110.2 (d), 118.3 (s), 127.3 (d), 128.1 (d), 128.4 (d), 142.0 (s), 142.1 (s), 144.8 (d), 145.0 (d), 146.26 (d), 146.29 (d), 149.8 (s), 149.9 (s), 151.8 (s), 157.00 (s), 157.01 (s), 157.03 (s), 157.06 (s), 157.07 (s), 163.1 (s), 163.2 (s), 163.3 (s), 163.9 (s), 168.47 (s), 168.49 (s), 168.7 (s), 169.5 (s), 170.9 (s), 171.1 (s), 171.38 (s), 171.45 (s), 171.48 (s), 171.66 (s), 171.72 (s), 173.3 (s), 173.4 (s), 175.1 (s), 176.7 (s), 176.8 (s).

**4.5. AZT (minor species indicated with appended “m”)**

^1^H NMR (700 MHz, d_6_-DMSO): *δ* = 1.468m (s, 3H) & 1.471 (s, 3H), 1.477m (s, 3H) & 1.480 (s, 3H), 1.414 (d, 3H, *J* = 6.18 Hz), 3.705 (m), 4.486 (dd, *J* = 8.02, 2.65 Hz), 6.945, 6.942, 6.939, & 6.931 (4 × s), 9.321m (d, *J* = 8.03 Hz) & 9.333 (d, *J* = 8.03 Hz). ^13^C NMR (176 MHz, d_6_-DMSO): *δ* = 17.98 (qt) & 17.98m (qt), 23.71 (qt) & 23.71m (qt), 23.79 (qt) & 23.79m (qt), 56.92 (d), 60.30 (d), 82.26m (s) & 82.31 (s), 111.00m (d) & 111.06 (d), 133.81 (v br s), 145.40 (v br s), 160.29 (br s), 161.61 (s) & 161.62m (s), 169.94m (br s) & 170.01 (br s), 172.24 (br s) & 172.26m (br s).

**4.6. AZT–BB linker (minor species indicated with appended “m”)**

^1^H NMR (700 MHz, d_6_-DMSO): *δ* = 1.12–1.17 (m, 2H), 1.17–1.23 (m, 2H), 1.34–1.40 (m, 2H), 1.37–1.43 (m, 2H), 1.417 (s, 3H), 1.425 (s, 3H), 1.425 (d, 3H, *J* = 6.18 Hz), 3.070 (ho m, 2H), 3.342 (t, 2H, *J* = 7.09 Hz), 3.720 (qtd, *J* = 6.14, 2.64 Hz), 4.540 (dd, *J* = 8.04, 2.63 Hz), 6.995, 6.989, 6.983, & 6.980 (4 × s), 6.991, 6.990, & 6.989 (3 × s, 2H), 7.296 (t, *J* = 5.90 Hz), 9.520 (d, *J* = 8.07 Hz). ^13^C NMR (176 MHz, d_6_-DMSO): *δ* = 17.97 (qt), 23.90 (qt) & 24.01m (qt), 24.17 (qt) & 24.21m (qt), 25.70m (t) & 25.73 (t), 25.83 (t) & 25.85m (t), 27.86 (t) & 27.88m (t), 28.93 (t), 36.96 (t), 38.51 (t), 57.00 (d), 60.20 (d), 83.92 (s), 111.25 (d), 134.44 (d) & 134.45m (d) & 134.46m (d), 135.13 (v br s), 146.91 (v br s), 160.82 (br s), 161.69 (s), 169.77 (br s), 171.08 (s) & 171.10m (s), 172.64 (s).

**4.7. AZT–R6 (minor species indicated with appended “m”)**

^1^H NMR (500 MHz, d_6_-DMSO): *δ* = 1.12–1.24 (ol m), 1.42 (d, *J* = 6.2 Hz), 1.33–1.44 (ol m), 1.44–1.60 (ol m), 1.62–1.75 (ol m), 2.46m (dd, *J* = 10.6 and 3.7 Hz), 2.88m (dd, *J* = 13.0 and 8.5 Hz), 2.97–3.24 (ol m), 3.33 (t, 2H, *J* = 6.8 Hz), 3.76 (dt, *J* = 6.2 and 2.5 Hz), 3.83 (br qt, *J* = 5.8 Hz), 4.02 (ddd, *J* = −16.7, 8.9, and 4.0 Hz), 4.19–4.30 (ol m), 4.34 (br qt, *J* = 6.5 Hz), 4.39 (dist qt, *J* = 7.2 Hz), 4.52 (dd, *J* = 8.0 and 2.4 Hz), 6.81 (s), 7.22 (t, *J* = 5.7 Hz), 7.26 (br s), 7.50–7.69 (ol m), 7.71 (br t, *J* = 6.2 Hz), 7.76 (br t, *J* = 5.8 Hz), 7.82 (br t, *J* = 5.9 Hz), 7.83 (br t, *J* = 5.9 Hz), 8.01–8.29 (ol m), 8.59 (br d, *J* = 6.9 Hz), 9.00m (d, *J* = 8.0 Hz), 9.41 (br d, *J* = 8.0 Hz). ^13^C NMR (125 MHz, d_6_-DMSO): *δ* = 18.0 (qt), 24.0 (qt), 24.1 (t), 24.3 (qt), 24.94 (t), 24.97 (t), 25.0 (t), 25.1 (t), 25.77 (t), 25.8 (t), 26.9 (t), 28.5 (t), 28.9 (t), 29.1 (t), 29.2 (t), 29.3 (t), 32.8 (t), 33.1 (t), 35.7 (t), 35.8 (t), 38.2 (t), 38.5 (t), 40.2 (t), 40.5 (t), 40.6 (t), 51.8 (d), 52.0 (d), 52.2 (d), 52.3 (d), 52.4 (d), 52.5 (d), 52.6 (d), 57.0 (d), 60.4 (d), 83.1 (t), 110.4 (d), 141.7 (s), 141.8 (s), 150.3 (s), 156.9 (s), 156.9 (s), 156.90 (s), 156.93 (s), 156.95 (s), 162.30 (s), 162.33 (s), 162.7 (s), 168.4 (s), 168.8 (s), 171.0 (s), 171.3 (s), 171.40 (s), 171.43 (s), 171.46 (s), 171.58 (s), 171.63 (s), 173.2 (s), 175.1 (s), 176.7 (s), 176.8 (s).

## **4.8. AMO**

^1^H NMR (500 MHz, d_6_-DMSO): *δ* = 1.40 (s, 3H), 1.51 (s, 3H), 4.02 (s, 1H), 4.73 (s, 1H), 5.35 (d, 1H, *J* = 4.0 Hz), 5.44 (m, 1H), 6.73 (XX’ part of AA’XX’, 2H, *J*_L+S_ = 8.6 Hz), 7.22 (AA’ part of AA’XX’, 2H, *J*_L+S_ = 8.7 Hz), 8.90 (br s, 1H), 9.50 (ex br s).

## **4.9. AMO–SMCC**

^1^H NMR (500 MHz, d_6_-DMSO): *δ* = 0.94–0.82 (m), 1.26 (tt, *J* = 12.8 and 3.1 Hz), 1.42 (s, 3H), 1.55 (s, 3H), 1.57–1.45 (m), 1.77–1.57 (m), 2.26 (tt, 1H, *J* = 12.0 and 3.4 Hz), 3.23 (d, *J* = 7.1 Hz), 4.19 (s, 1H), 5.39 (d, 1H, *J* = 4.1 Hz), 5.53–5.49 (ol m, 2H), 6.78 (XX’ part of AA’XX’, 2H, *J*_L+S_ = 8.6 Hz), 7.00 (s), 7.18 (AA’ part of AA’XX’, 2H, *J*_L+S_ = 8.6 Hz), 9.35 (v br s, 1H), 13.13 (ex br s).

## **4.10. AMO–R6**

^1^H NMR (500 MHz, d_6_-DMSO): *δ* = 0.89 (br qt, 2H, *J* = 12.1 Hz), 1.30–1.21 (ol m), 1.42 (s, 3H), 1.55 (s, 3H), 1.59–1.44 (ol m), 1.80–1.59 (ol m), 2.26 (br tt, *J* = 11.8 and unres Hz), 2.54 (s, 2H), 3.25–2.99 (ol m), 3.67–3.57 (ol m, 3H), 3.82 (br t, 1H, *J* = 6.1 Hz), 4.03 (ddd, 1H, *J* = 17.8, 8.8, and 4.0 Hz), 4.12 (s, 1H), 4.30–4.19 (ol m, 4H), 4.34 (dist br qt, 1H), 4.39 (br qt, 1H, *J* = 7.2 Hz), 5.37 (d, 1H, *J* = 4.1 Hz), 5.51–5.46 (ol m, 2H), 6.69 (XX’ part of AA’XX’, 2H, *J*_L+S_ = 8.6 Hz), 7.17 (AA’ part of AA’XX’, 2H, *J*_L+S_ = 8.7 Hz), 7.27 (br d, 1H, *J* = 5.3 Hz), 7.75–7.68 (ol m, 2H), 7.77 (br t, 1H, *J* = 5.4 Hz), 7.82 (br t, 1H, *J* = 5.1 Hz), 7.88 (br t, 1H, *J* = 5.3 Hz), 7.91 (br s, 1H), 8.09 (br t, 1H, *J* = 7.7 Hz), 8.14 (br t, 1H, *J* = 7.5 Hz), 8.19 (br d, 1H, *J* = 7.0 Hz), 8.23 (d, 1H, *J* = 8.0 Hz), 8.43 (br s, 1H), 8.59 (br d, 1H, *J* = 7.0 Hz), 8.80 (br s, 1H), 9.44 (s, 1H), 13.29 (ex br s).

## **4.11. ERT**

^1^H NMR (500 MHz, CD_3_OD): *δ* = 1.20 (d, 3H, *J* = 7.2 Hz), 1.28 (d, 3H, *J* = 6.3 Hz), 1.74 (dt, 1H, *J* = 13.5 and 7.1 Hz), 2.68 (ddd, 1H, *J* = 13.5, 9.0, and 7.9 Hz), 2.92 (dd, 1H, *J* = 11.7 and 5.6 Hz), 3.20 (dd, 1H, *J* = 7.1 and 2.6 Hz), 3.31–3.26 (ol m), 3.70–3.62 (m, 1H), 3.87 (dd, 1H, *J* = 9.0 and 7.3 Hz), 4.09 (qn, 1H, *J* = 6.6 Hz), 4.11 (dd, 1H, *J* = 9.3 and 2.5 Hz), 7.31 (ddd, 1H, *J* = 8.3, 7.5, and 0.5 Hz), 7.70 (ddd, 1H, *J* = 7.7, 1.6, and 1.1 Hz), 7.33 (ddd, 1H, *J* = 8.0, 2.3, and 1.1 Hz), 7.97 (t, 1H, *J* = 1.9 Hz), 8.04 (t, 1H, *J* = 1.9 Hz), 8.10 (t, 1H, *J* = 1.9 Hz).

## **4.12. ERT–SMCC**

^1^H NMR (500 MHz, CD_3_OD): *δ* = 1.14–0.94 (ol m), 1.26 (d, 3H, *J* = 7.2 Hz), 1.29 (d, 3H, *J* = 6.3 Hz), 1.53–1.19 (ol m), 1.86–1.57 (ol m), 2.14–2.07 (ol m), 2.20 (tt, 1H, *J* = 12.3 and 3.5 Hz), 2.57–2.46 (m, 1H), 2.82–2.74 (m, 1H), 3.37–3.24 (ol m), 3.60–3.53 (ol m, 2H), 3.91 (br t, 1H, *J* = 8.4 Hz), 4.15–4.07 (m, 1H), 4.32–4.18 (ol m, 3H), 4.54 (t, 1H, *J* = 8.1 Hz), 6.79 (br s, 1H), 7.40 (br t, 1H, *J* = 7.9 Hz), 7.75 (br d, 1H, *J* = 7.6 Hz), 7.81 (br d, 1H, *J* = 8.0 Hz), 8.23 (br s, 1H).

## **4.13. ERT–R6**

^1^H NMR (500 MHz, CD_3_OD): *δ* = 1.16–0.89 (ol m), 1,24 (d, 3H, *J* = 7.2 Hz), 1.29 (d, 3H, *J* = 6.2 Hz), 2.06–1.43 (ol m), 2.60–2.48 (ol m), 2.81–2.72 (m, 1H), 3.40–2.97 (ol m), 3.67–3.58 (m, 1H), 3.90–3.80 (m, 1H), 4.12 (qn, 1H, *J* = 6.3 Hz), 4.27–4.18 (ol m), 4.32–4.27 (ol m), 4.41–4.33 (ol m), 4.47–4.41 (ol m, 1H), 4.58 (t, 1H, *J* = 8.7 Hz), 4.65–4.59 (ol m, 1H), 7.44–7.26 (ol m), 7.67 (d, *J* = 7.5 Hz), 8.46 (br s), 8.63 (br s).

**5. LC/MS analysis of the β-lactam–peptide conjugates and their MICs on *B. subtilis* DSM10**

## **5.1. List of β-lactam–peptide conjugates**

**Table S3.** Characterization of β-lactam–peptide conjugates and their corresponding MICs against B. subtilis DSM10. The table lists the compounds with their corresponding calculated molecular ions, calculated charged ions, observed ions and MICs against B. subtilis DSM10 (shown as medians, n = 3). Legend for lost fragments: py, pridinyl; CO_2_, carbon dioxide; SO_3_, sulfur trioxide.^[2]^

| **Compound** | **Calcu-lated molecular ion**  **[Da]** | **Calcu-lated charged ion**  **[*m*/*z*, Da]** | **Observed ion**  **[*m*/*z*, Da]** | **MIC**  **[µg mL^−1^]** |
| --- | --- | --- | --- | --- |
| CTZ | 546.10 | – | – | 8 |
| CTZ-R1 | 1001.33 | 878.30 | 878.31 [M – py – CO_2_ – H + H^+^]^+^ | 1 |
| CTZ-R3 | 1313.53 | 1234.49 | 1234.49 [M – py – H + H^+^]^+^ | 0.06 |
| CTZ-R6 | 1781.84 | 851.90 | 851.91 [M – py – H + 2H^+^]^+2^ | 0.008 |
| CTZ-R9 | 2250.14 | 710.04 | 710.04 [M – py – CO_2_ + 3H^+^]^+3^ | 0.06 |
| CTZ-R12 | 2718.44 | 1320.71 | 1320.71 [M – py + 2H^+^]^+2^ | 0.06 |
| CTZ-K1 | 973.32 | 850.29 | 850.31 [M – py – CO_2_ – H + H^+^]^+^ | 1 |
| CTZ-K3 | 1229.51 | 1107.49 | 1107.48 [M – py – CO_2_ + H^+^]^+^ | 0.5 |
| CTZ-K6 | 1613.80 | 767.88 | 767.89 [M – py – H + 2H^+^]^+2^ | 0.02 |
| CTZ-K9 | 1998.08 | 938.53 | 938.53 [M – py – CO_2_ + 2H^+^]^+2^ | 0.008 |
| CTZ-K12 | 2382.37 | 1130.68 | 1130.68 [M – py – CO_2_ + 2H^+^]^+2^ | 0.02 |
| CTZ-(GR)3 | 1484.60 | 681.29 | 681.29 [M – py – CO_2_ – H + 2H^+^]^+2^ | 0.06 |
| CTZ-E6 | 1619.49 | 1619.49 | 1619.49 [M – H + H^+^]^+^ | > 64 |
| CTZ-R3K3 | 1697.82 | 787.90 | 787.90 [M – py – CO_2_ – H + 2H^+^]^+2^ | 0.008 |
| CTZ-(KR)3 | 1697.82 | 787.90 | 787.90 [M – py – CO_2_ – H + 2H^+^]^+2^ | 0.008 |
| CTZ-G6 | 1187.36 | 1064.33 | 1064.34 [M – py – CO_2_ – H + H^+^]^+^ | 8 |
| CTZ-yR6 | 1944.90 | 933.43 | 933.45 [M – py – H + 2H^+^]^+2^ | 0.03 |
| CTZ-yK6 | 1776.86 | 849.92 | 849.93 [M – py + 2H^+^]^+2^ | 0.02 |
| AZT | 435.05 | – | – | 512 |
| AZT-R1 | 890.28 | 810.33 | 810.34 [M – SO_3_ – H + H^+^]^+^ | 16 |
| AZT-R3 | 1202.49 | 1202.49 | 1202.50 [M – H + H^+^]^+^ | 1 |
| AZT-R6 | 1670.79 | 835.90 | 835.90 [M – H + 2H^+^]^+2^ | 0.06 |
| AZT-R9 | 2139.09 | 714.04 | 714.04 [M + 3H^+^]^+3^ | 0.5 |
| AZT-R12 | 2607.40 | 652.86 | 652.86 [M + 4H^+^]^+4^ | 1 |
| AZT-K1 | 862.28 | 782.32 | 782.34 [M – SO_3_ – H + H^+^]^+^ | 16 |
| AZT-K3 | 1118.47 | 1119.47 | 1119.46 [M + H^+^]^+^ | 8 |
| AZT-K6 | 1502.75 | 751.88 | 751.89 [M – H + 2H^+^]^+2^ | 0.25 |
| AZT-K9 | 1887.04 | 944.53 | 944.53 [M + 2H^+^]^+2^ | 0.1 |
| AZT-K12 | 2271.32 | 1136.67 | 1136.67 [M + 2H^+^]^+2^ | 0.06 |
| AZT-(GR)3 | 1373.55 | 1373.55 | 1373.57 [M – H + H^+^]^+^ | 1 |
| AZT-E6 | 1508.44 | 1428.48 | 1428.49 [M – SO_3_ – H + H^+^]^+^ | > 64 |
| AZT-R3K3 | 1586.77 | 793.89 | 793.89 [M – H + 2H^+^]^+2^ | 0.06 |
| AZT-(KR)3 | 1586.77 | 793.89 | 793.90 [M – H + 2H^+^]^+2^ | 0.06 |
| AZT-G6 | 1076.31 | 996.35 | 996.37 [M – SO_3_ – H + H^+^]^+^ | 64 |
| AZT-yR6 | 1833.85 | 917.43 | 917.44 [M – H + 2H^+^]^+2^ | 1 |
| AMO | 365.10 | – | – | 0.1 |
| AMO-R6 | 1641.82 | 821.41 | 821.42 [M – H + 2H^+^]^+2^ | 0.06 |
| AMO-yR6 | 1804.88 | 902.95 | 902.95 [M – H + 2H^+^]^+2^ | – |
| ERT | 475.14 | – | – | 0.5 |
| ERT-R6C | 1751.86 | 876.43 | 876.44 [M – H + 2H^+^]^+2^ | < 0.02 |
| ERT-yR6 | 1914.92 | 639.31 | 639.32 [M + 3H^+^]^+3^ | – |

## **5.2. HPLC profile and mass spectrum of CTZ-R6**

**Figure S6.** HPLC profile, mass spectrum and chemical structure of CTZ-R6. Assignments: 851.91 [M − py − H + 2H^+^]^+2^, 829.92 [M − py − CO_2_ − H + 2H^+^]^+2^, 891.93 [M + 2H^+^]^+2^. Legend for lost fragments: py, pridinyl; CO_2_, carbon dioxide.


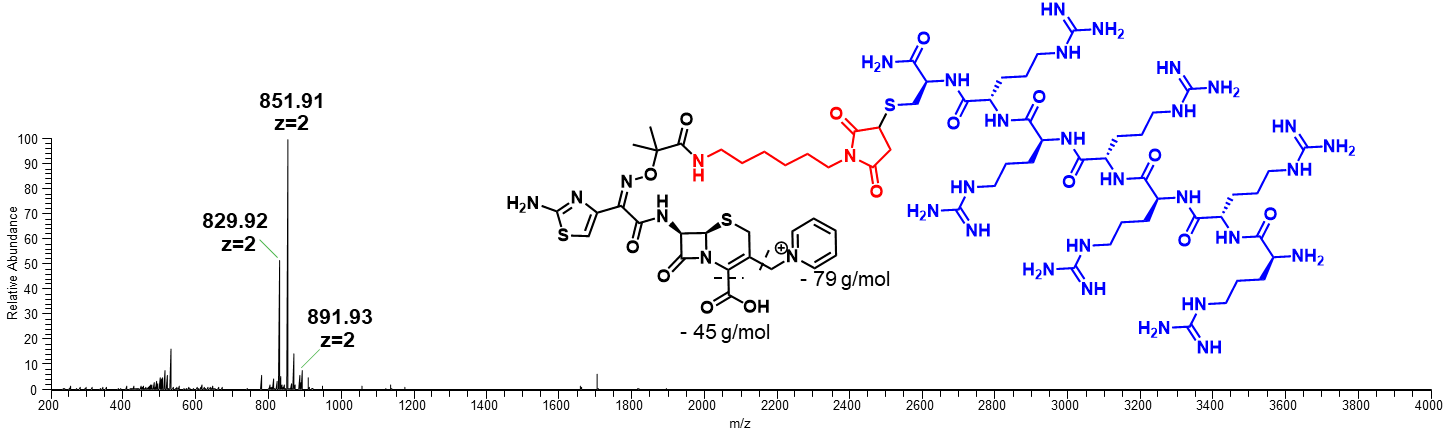

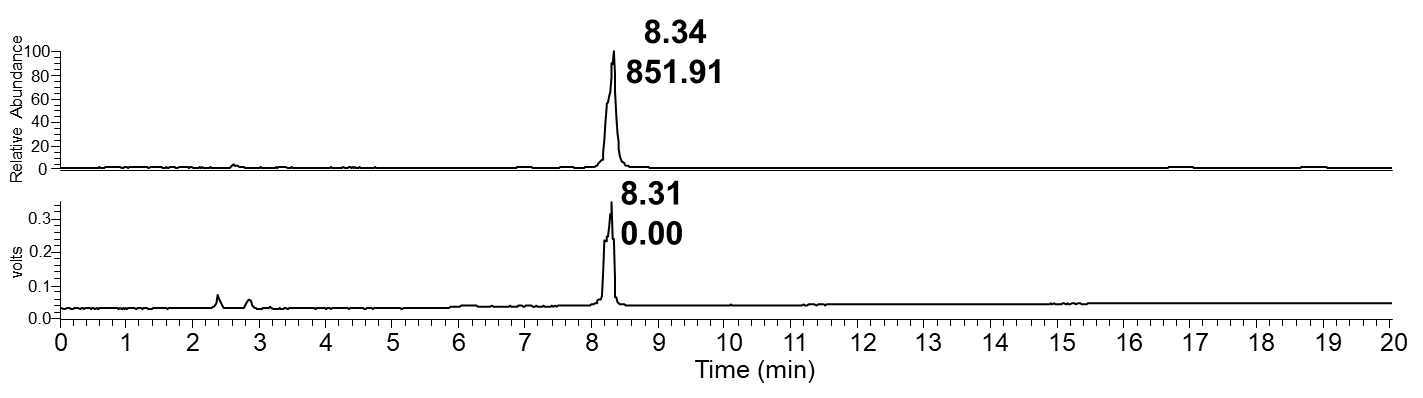


## **5.3 HPLC profile and mass spectrum of AZT-R6**

**Figure S7.** Mass spectrum and chemical structure of AZT-R6. Assignments: 835.90 [M − H + 2H^+^]^+2^, 1670.80 [M − H + H^+^]^+^.


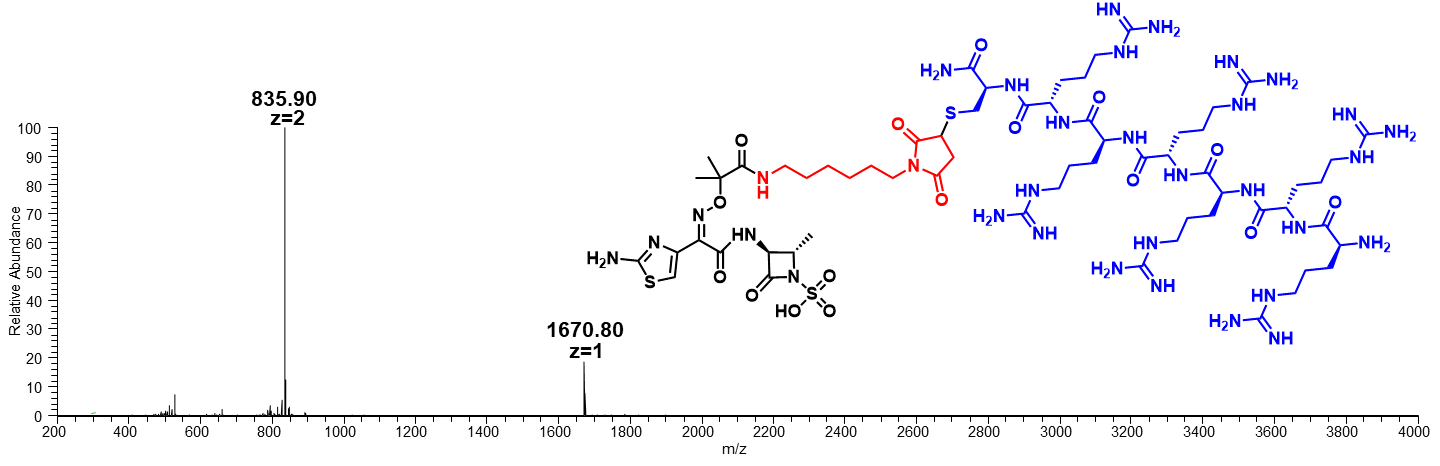

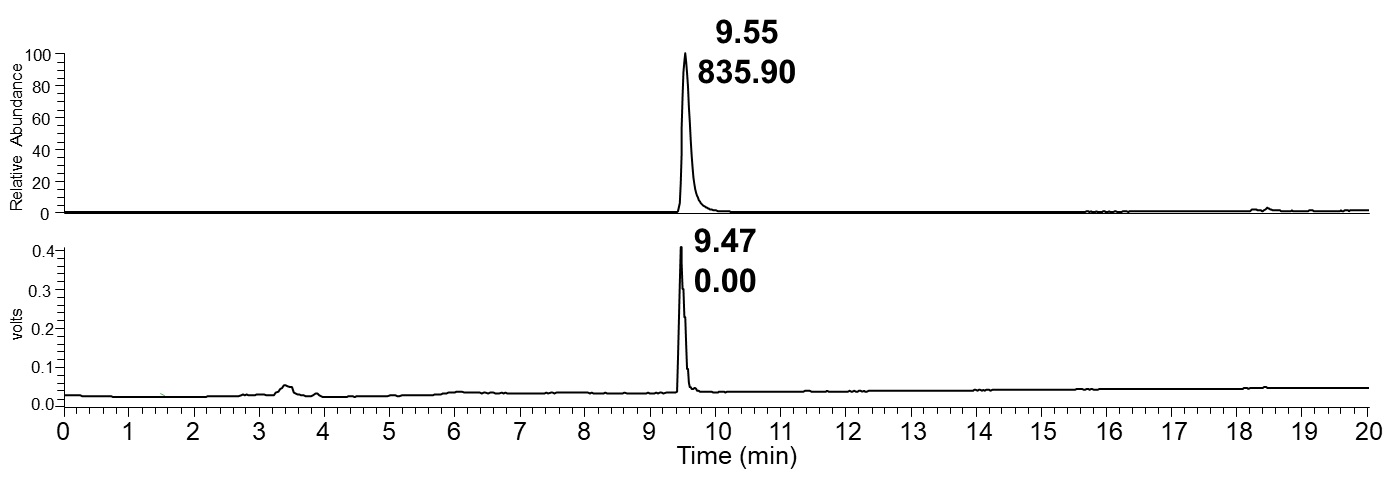


## **5.4. HPLC profile and mass spectrum of AMO-R6**

**Figure S8.** HPLC profile, mass spectrum and chemical structure of AMO-R6. Assignments: 821.42 [M − H + 2H^+^]^+2^.


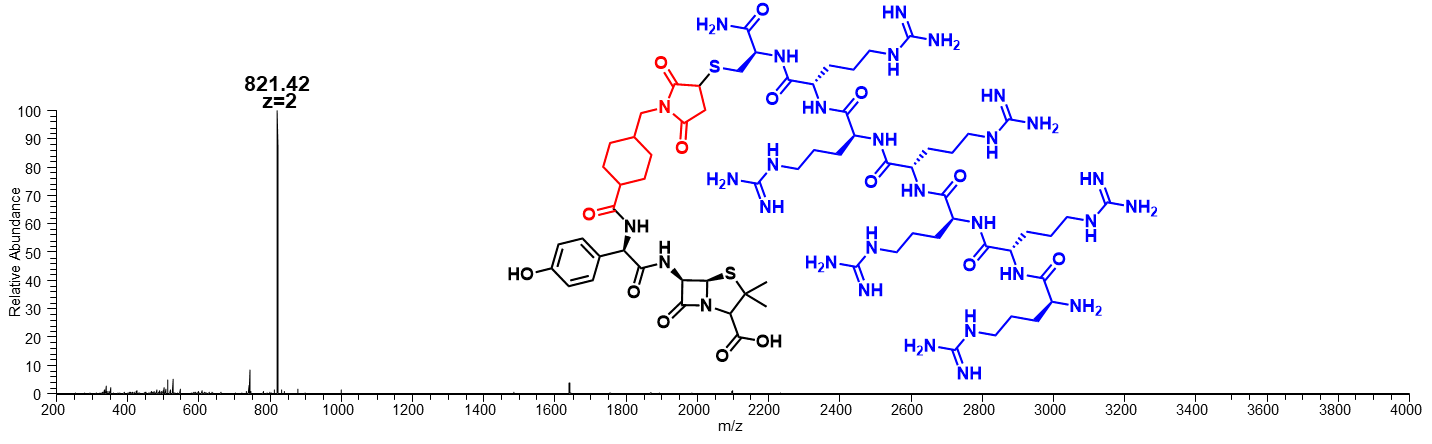

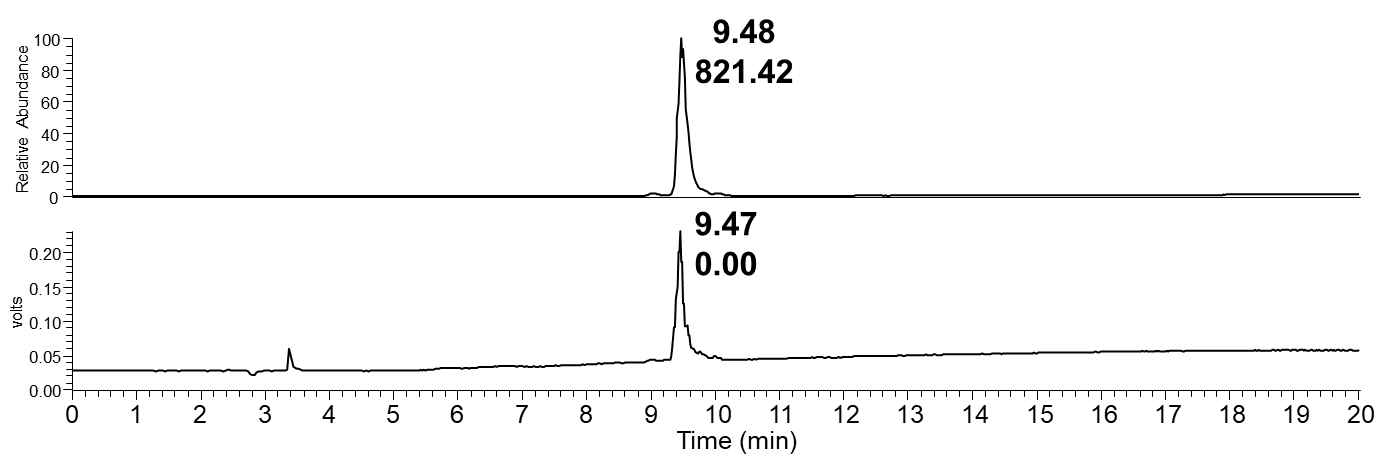


## **5.5. HPLC profile and mass spectrum of ERT-R6**

**Figure S9.** HPLC profile, mass spectrum and chemical structure of ERT-R6. Assignments: 876.44 [M − H + 2H^+^]^+2^, 1752.87 [M + H^+^]^+^.


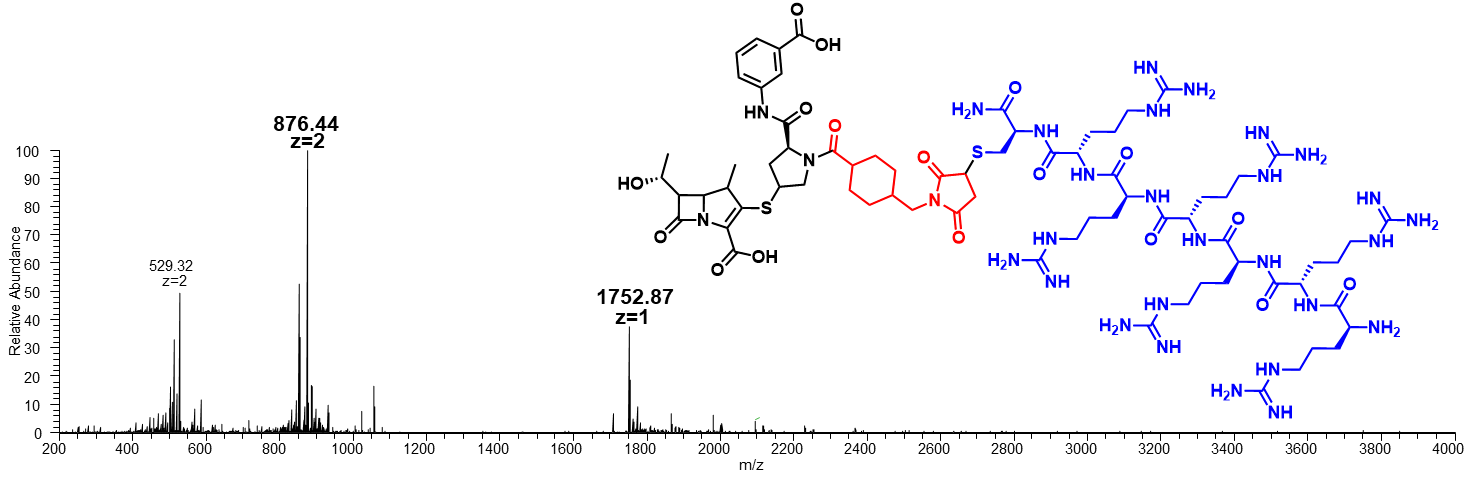

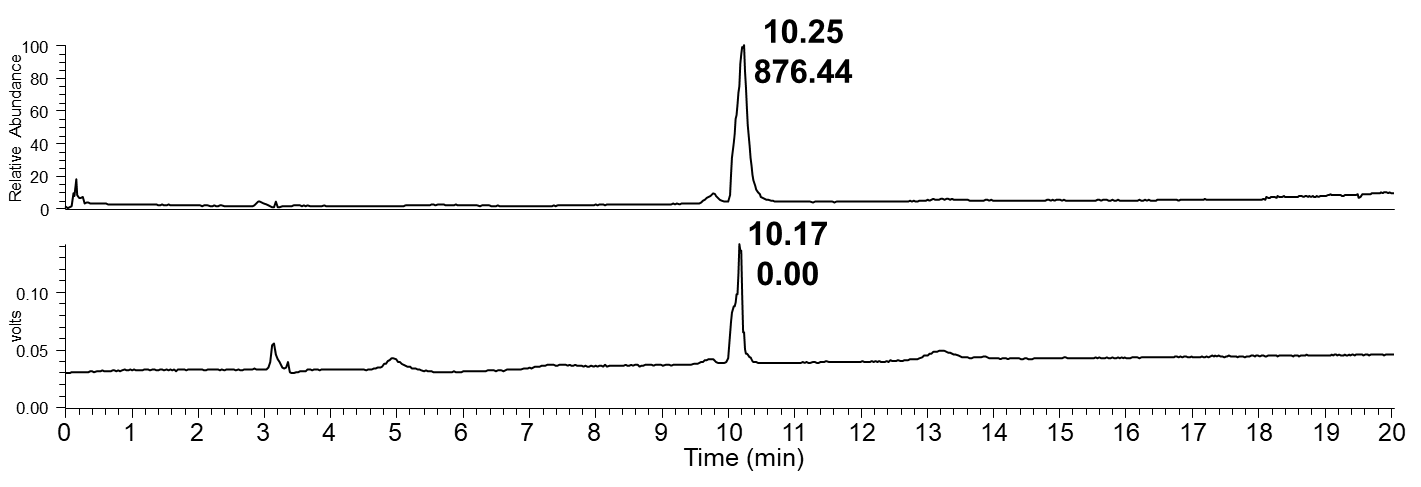


# **
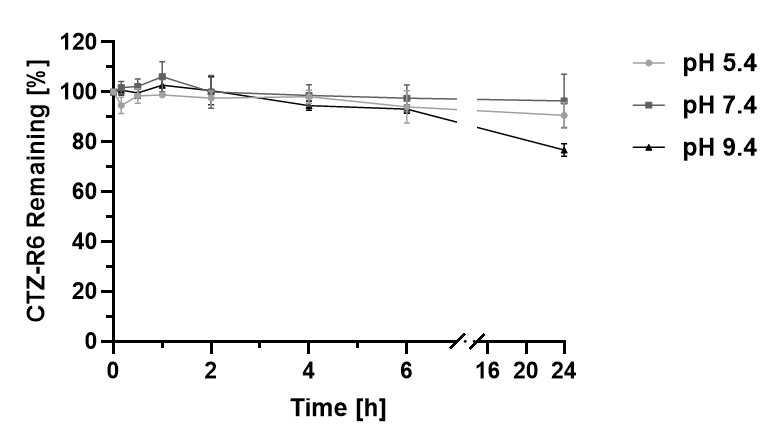
6. Stability assessment of CTZ-R6**

**Figure S10.** Stability of CTZ-R6 in PBS at different pH values. CTZ-R6 showed high stability in PBS at all tested pH conditions over 24 h.

**7. Antimicrobial activities of AZT–peptide conjugates**

**Figure S11.** Antimicrobial activities of AZT–peptide conjugates against (A) B. subtilis DSM 10 and (B) A. bohemicus DSM 100419. Similar to the CTZ–peptide conjugates, increasing the peptide charge results in increased antimicrobial activity against both bacterial strains. Data is shown as medians (n = 3).

**
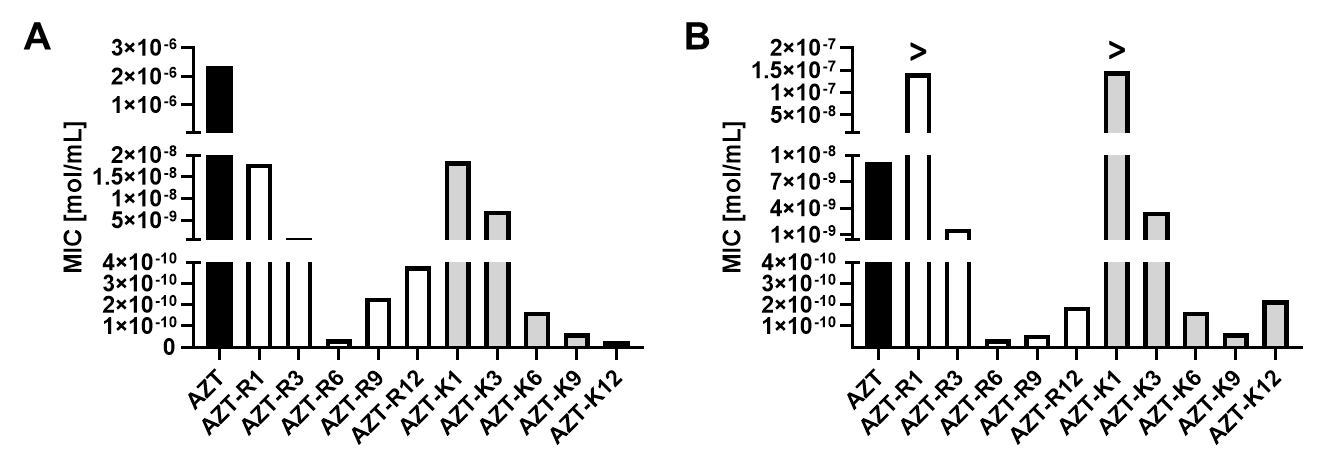
****8. Antimicrobial activities of individual components of CTZ-R6**

**Table S4.** Antimicrobial activities of the individual components of CTZ-R6 against different Gram-positive strains. Data is shown as medians (n = 3).

|  | **MIC [µg mL^−1^]** | | | |
| --- | --- | --- | --- | --- |
| **Bacterial strain** | **R6C** | **BB linker** | **CTZ + R6C** | **CTZ–BB linker** |
| *B. subtilis* DSM 10 | > 64 | > 64 | 12 | 24 |
| *E. faecalis* ATCC 29212 | > 64 | > 64 | > 64 | 1 |
| *E. casseliflavus* ATCC 700327 | > 64 | > 64 | > 64 | > 64 |

# **9. Antimicrobial activities of β-lactam–peptide conjugates against staphylococci**

**Table S5.** Antimicrobial activities of CTZ-R6 and its originator against different staphylococci. Data is shown as median (n = 3).

|  | **MIC [µg mL^−1^]** | |
| --- | --- | --- |
| **Bacterial strain** | **CTZ** | **CTZ-R6** |
| *S. saprophyticus*  ATCC 15305 | 32 | 0.02 |
| *S. aureus*  ATCC 25923 (MSSA) | 16 | 4 |
| *S. aureus* LAC* *lux*  USA300 (MRSA) | 16 | 4 |
| *S. aureus*  NCTC 10442 (MRSA) | 16 | 2 |

# **10. Testing of synergism**

**Table S6.** Synergy of different antibiotic combinations against enterococci. Data is shown as median (n = 3). Synergy was defined as a FIC index ≤ 0.5, indifference as a fractional inhibitory concentration (FIC) index between > 0.5 and 4 and antagonism as a FIC index > 4.^[3]^

|  | **FIC index** | | |
| --- | --- | --- | --- |
| **Bacterial strain** | **CTZ + CTZ-R6** | **CTZ + VAN** | **CTZ-R6 +VAN** |
| *E. faecalis* ATCC 51299 | 0.75 | 0.13 | 0.14 |
| *E. faecium* UL 602570* | 1.13 | 0.63 | 0.69 |
| * Clinical isolate. |  |  |  |

# **11. Antimicrobial activities of β-lactam–peptide conjugates and β-lactam inhibitor combinations against Gram-negative bacteria**

## **11.1. MICs of the combination of AZT-R6 and avibactam (AVI)**

**Table S7.** Antimicrobial activities of combinations of AZT, AZT-R6 and AVI against different Gram-negative bacteria. Data is shown as medians (n = 3).

|  | **MIC [µg mL^−1^]** | | | | |
| --- | --- | --- | --- | --- | --- |
| **Bacterial strain**  (β-lactamase) | **AZT** | **AZT + AVI** | **AZT-R6** | **AZT-R6 + AVI** | **AVI** |
| *E. coli* ATCC 25922 (–) | 0.06 | < 0.03 | 0.09 | < 0.03 | 16 |
| *K. pneumoniae* ATCC 700603 (SHV-18) | 64 | 0.1 | 24 | < 0.03 | > 64 |
| *K. pneumoniae* BL809453*  (KPC) | > 64 | 0.25 | > 64 | 0.1 | > 64 |
| *K. pneumoniae* 06841/Nr.45* (Oxa48) | > 64 | 0.1 | > 64 | < 0.03 | > 64 |
| *A. baumannii* SC300007*  (Oxa23) | 64 | > 64 | 64 | > 64 | > 64 |

* Clinical isolate.

## **11.2. MICs of the combination of AMO-R6 and clavulanic acid (CLV)**

**Table S8.** Antimicrobial activities of combinations of AMO, AMO-R6 and CLV against different Gram-negative bacteria. Data is shown as medians (n = 3).

|  | **MIC [µg mL^−1^]** | | | | |
| --- | --- | --- | --- | --- | --- |
| **Bacterial strain**  (β-lactamase) | **AMO** | **AMO + CLV** | **AMO-R6** | **AMO-R6 + CLV** | **CLV** |
| *E. coli* ATCC 25922 (–) | 6 | 1.5 | 0.09 | 0.06 | 32 |
| *K. pneumoniae* ATCC 700603 (SHV-18) | > 64 | 4 | > 64 | 2 | 64 |
| *K. pneumoniae* BL809453* (KPC) | > 64 | > 64 | > 64 | > 64 | > 64 |
| *K. pneumoniae* 06841/Nr.45* (Oxa48) | > 64 | > 64 | > 64 | > 64 | > 64 |
| *A. baumannii* SC300007*  (Oxa23) | > 64 | > 64 | > 64 | > 64 | > 64 |

* Clinical isolate.

**
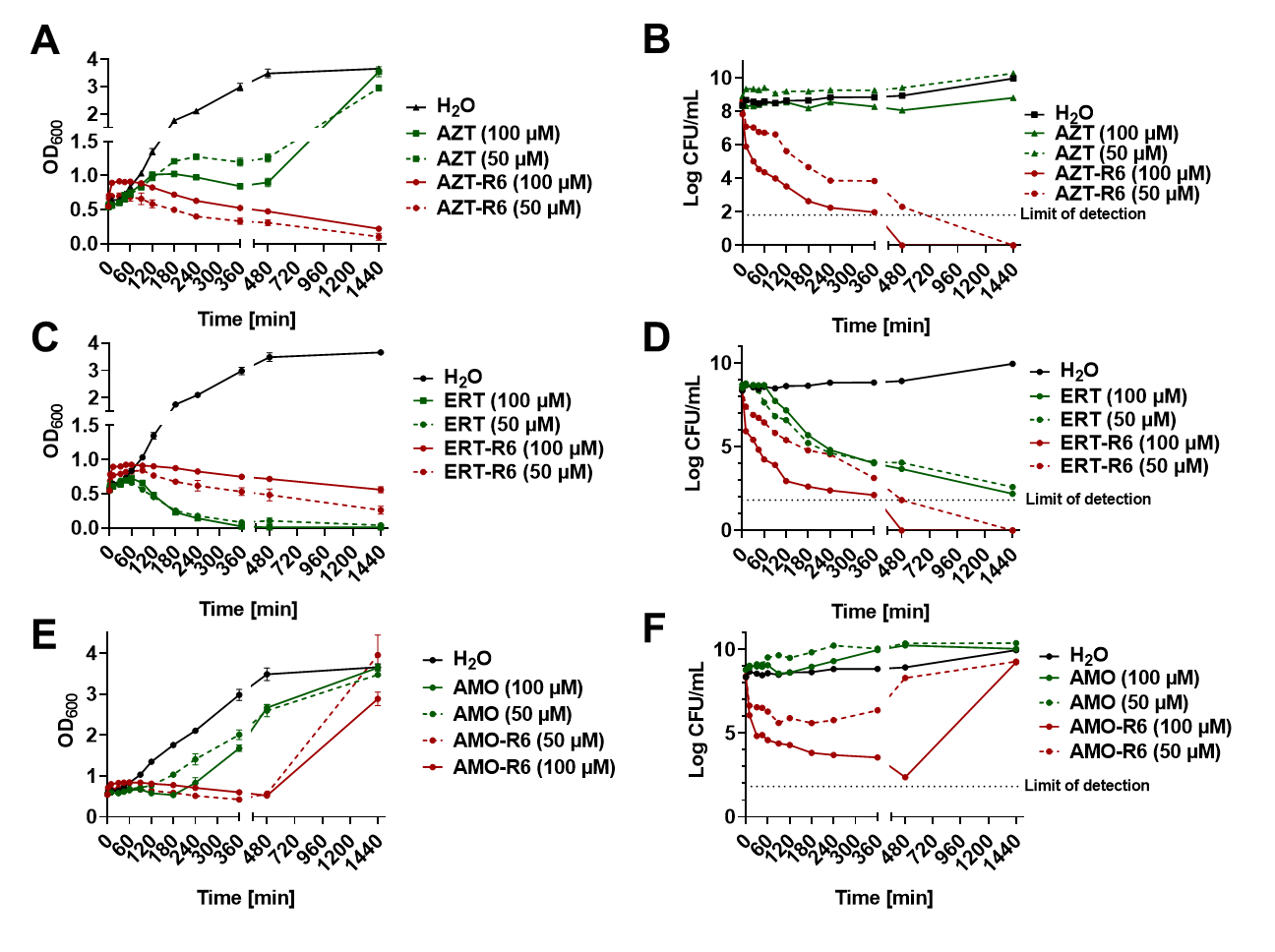
12. Time-kill studies**

**Figure S12.** Time-kill studies of β-lactam conjugates and their originators against B. subtilis. (A,C,E) depict OD_600_ measurements and (B,D,F) depict CFU mL^−1^ measurements over 24 h. Data is shown as mean (n = 3).

**13. PBP binding profiles**

## **
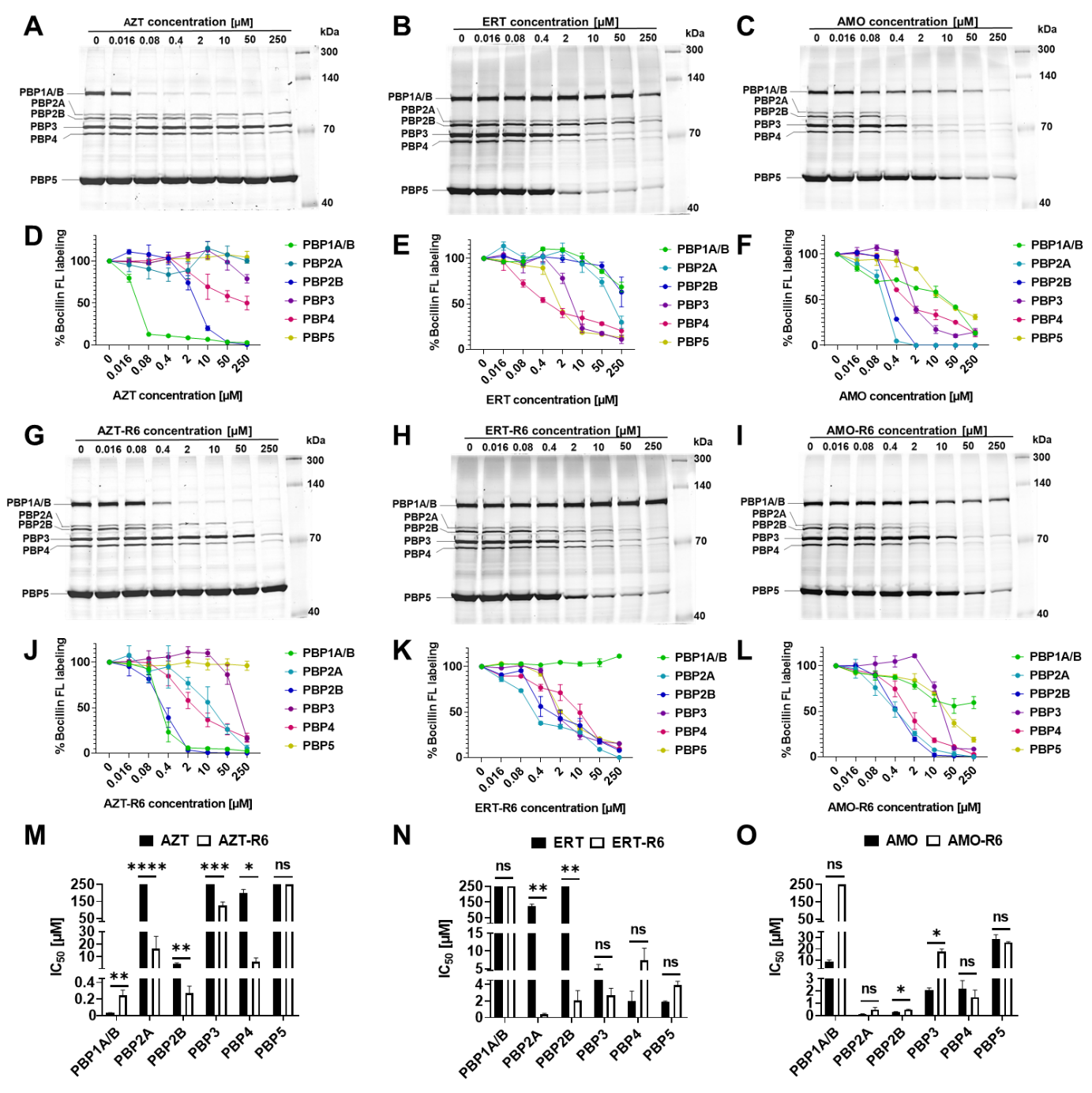
13.1. Binding profiles of β-lactam–peptide conjugates and parents to *B. subtilis* PBPs**

**Figure S13.** PBP binding profile of β-lactam–peptide conjugates and parents to B. subtilis PBPs. Representative SDS-PAGE gel images and quantitative analysis for inhibition of PBPs by (A,D) AZT, (B,E) ERT, (C,F) AMO, (G,J) AZT-R6, (H,K) ERT-R6 and (I,L) AMO-R6 are depicted. (M–O) Comparison of IC_50_ values of conjugates and their parent β-lactam antibiotics. Data is shown as mean ± SD (n = 3).

##
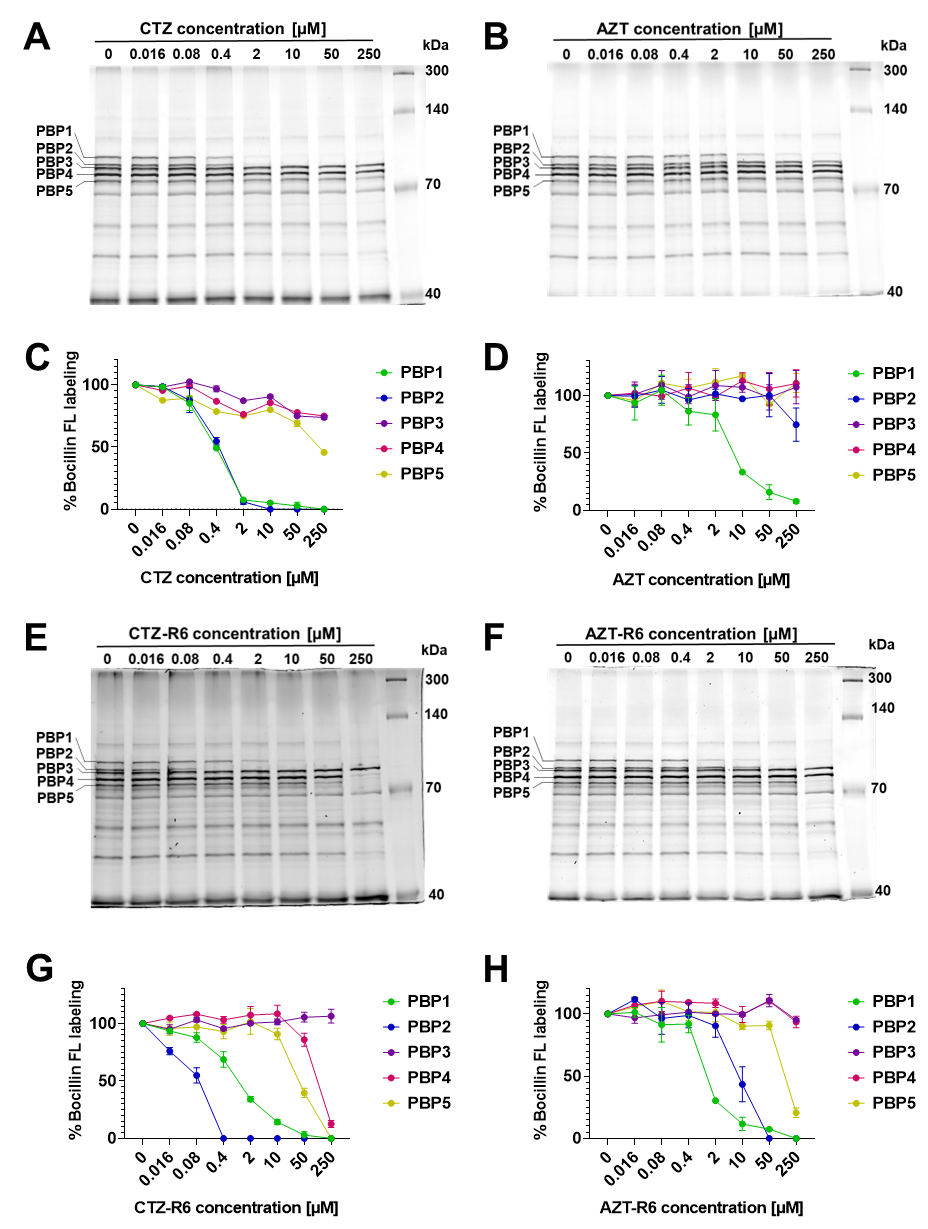
**13.2. Binding profiles of β-lactam–peptide conjugates and originators to *E. faecalis* PBPs**

**Figure S14.** PBP binding profile of β-lactam–peptide conjugates and parents to E. faecalis PBPs. Representative SDS-PAGE gel images and quantitative analysis for inhibition of PBPs by (A,C) CTZ, (B,D) AZT, (E,G) CTZ-R6 and (F,H) AZT-R6 are shown. Data is shown as mean ± SD (n = 3).

**
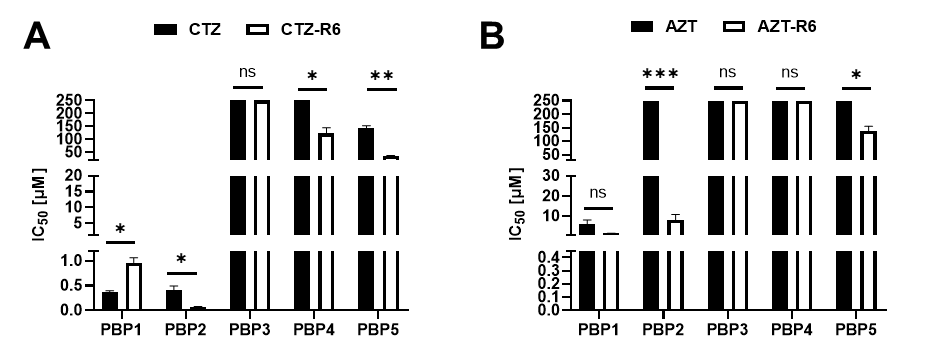
13.3. IC_50_ values of PBP (*E. faecalis)* binding by β-lactam–peptide conjugates**

**Figure S15.** Comparison of IC_50_ values of the β-lactam–peptide conjugates and their originators. (A) CTZ and CTZ-R6 and (B) AZT and AZT-R6. Data is shown as mean ± SD (n = 3).

# **14. Cytotoxicity evaluation**

## **
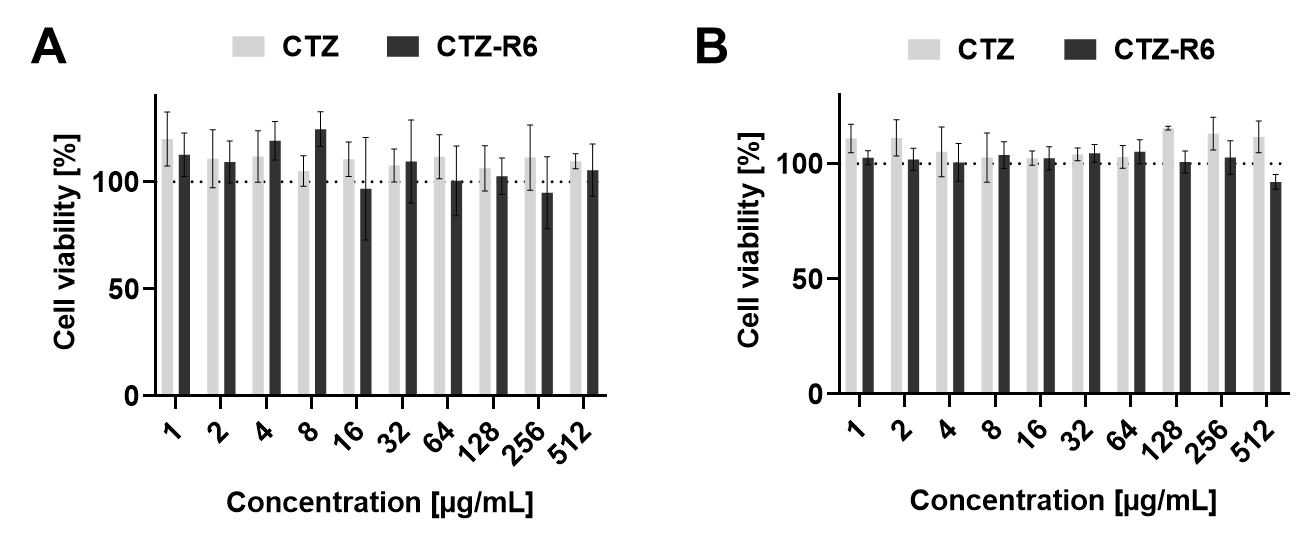
14.1. *In vitro* cytotoxicity**

**Figure S16.** Cytotoxicity evaluation of CTZ and CTZ-R6. (A) Cytotoxicity against the HEP-G2 cell line (liver) and (B) cytotoxicity against the HEK-293 cell line (kidney). Data is shown as mean ± SD (n = 3).

## **
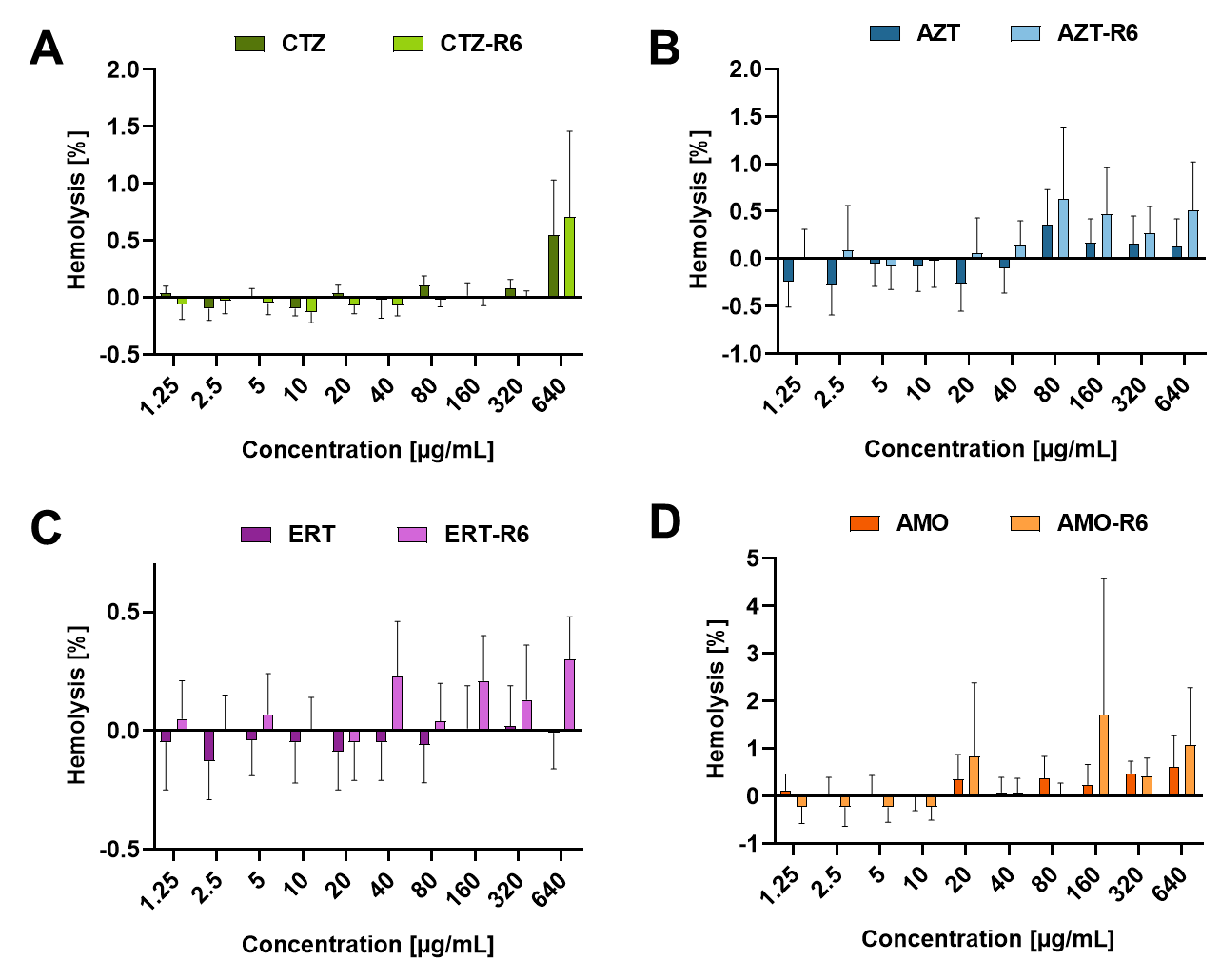
14.2. Hemolysis**

**Figure S17.** Hemolysis studies of β-lactam conjugates and their originators. (A) CTZ and CTZ-R6, (B) AZT and AZT-R6, (C) ERT and ERT-R6 and (D) AMO and AMO-R6. Data is shown as mean ± SD (n = 3).

**15. Pharmacokinetics**

**
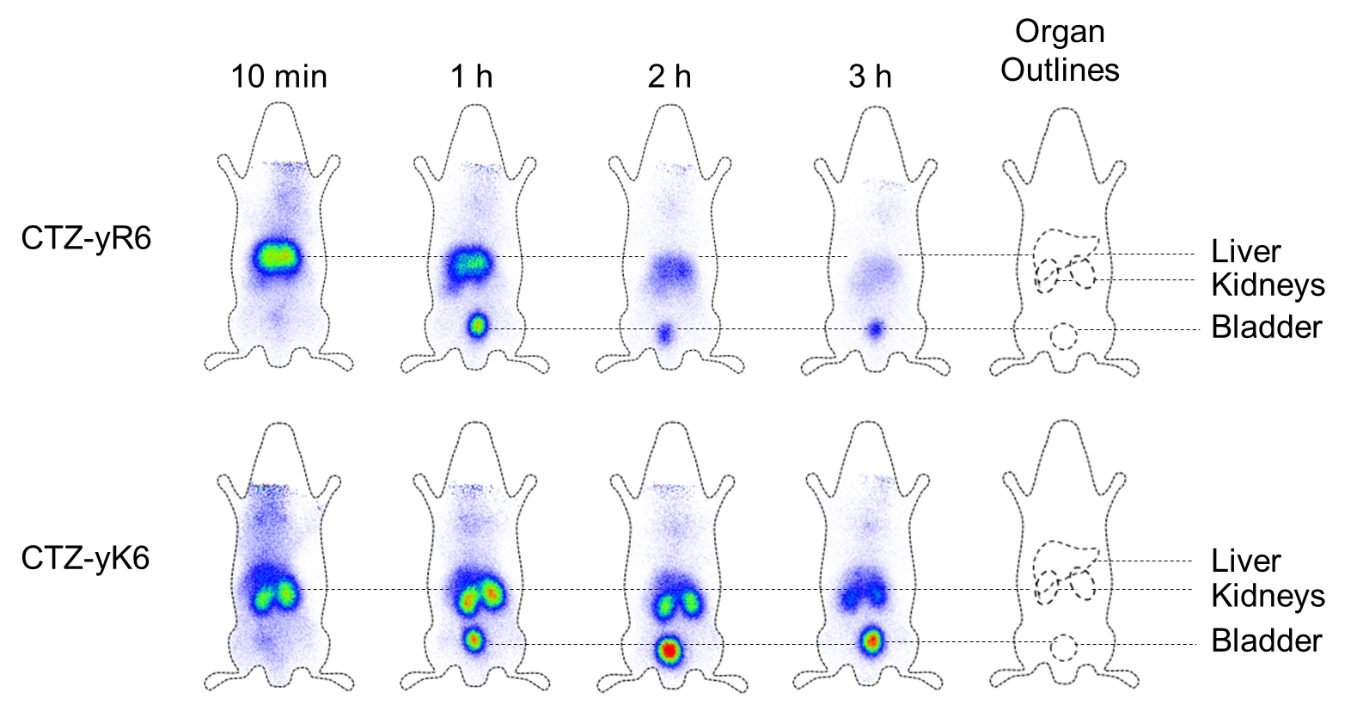
15.1. Biodistribution of CTZ-R6 and CTZ-K6 in mice**

**Figure S18.** Scintigraphic images of CTZ-yR6 and CTZ-yK6 in Swiss mice. While CTZ-yR6 is mainly accumulated in the liver, CTZ-yK6 is eliminated by the kidneys.

## **
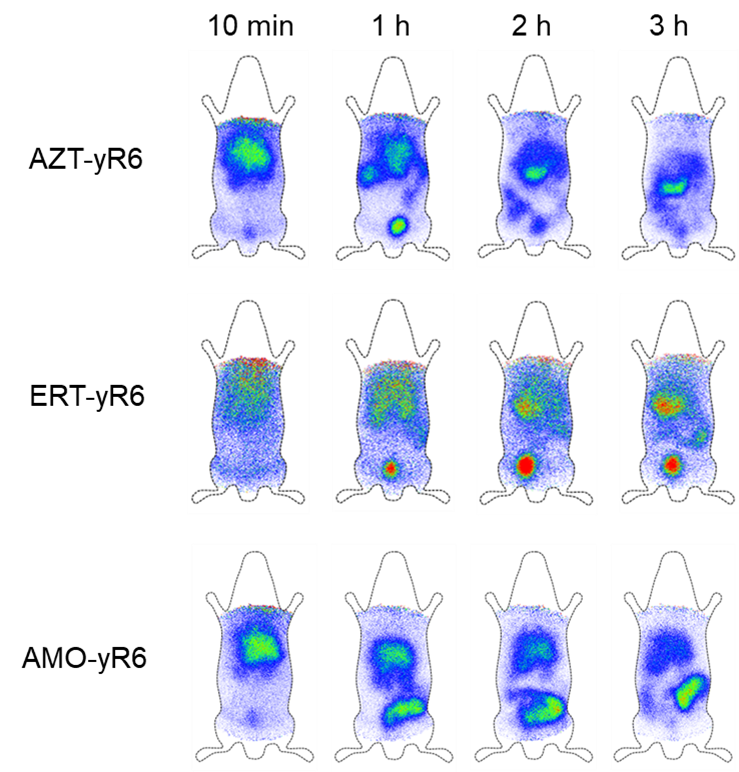
15.2. Biodistribution of β-lactam–peptide conjugates in rats**

**Figure S19.** Scintigraphic images of representative β-lactam–peptide conjugates in Wistar rats. 10 min post intravenous injection, the β-lactam–hexa-arginine conjugates showed an accumulation in the liver. Depending on the conjugate, further organs were addressed at later time points.

# **16. References**

[1] EUCAST, EUCAST System for Antimicrobial Abbreviations. **2018**.

[2] a) Q. Ye, W. Ding, F. Rinaldi, Y. Huang, S. A. Miller, M. Bolgar, *J Pharm Biomed Anal* **2016**, *124*, 358, https://doi.org/10.1016/j.jpba.2016.03.017; b) X.-M. Chong, C.-Q. Hu, *Chromatographia* **2008**, *68* (9-10), 759, https://doi.org/10.1365/s10337-008-0790-4; c) M. R. Siddiqui, A. A. H. Hakami, S. M. Wabaidur, Z. A. Alothman, M. A. Khan, F. M. Husain, *Food Science and Technology* **2021**, *41*, 225, https://doi.org/10.1590/fst.07020.

[3] E. Wang, M. S. Braun, M. Wink, *Molecules* **2019**, *24* (16), https://doi.org/10.3390/molecules24162968.
